# Design, synthesis, *in silico* studies and *in vitro* evaluation of isatin-pyridine oximes hybrids as novel acetylcholinesterase reactivators

**DOI:** 10.1080/14756366.2021.1916009

**Published:** 2021-06-21

**Authors:** Daniel A. S. Kitagawa, Rafael B. Rodrigues, Thiago N. Silva, Wellington V. dos Santos, Vinicius C. V. da Rocha, Joyce S. F. D. de Almeida, Leandro B. Bernardo, Taynara Carvalho-Silva, Cintia N. Ferreira, Angelo A. T. da Silva, Alessandro B. C. Simas, Eugenie Nepovimova, Kamil Kuča, Tanos C. C. França, Samir F. de A. Cavalcante

**Affiliations:** a Laboratory of Molecular Modelling Applied to Chemical and Biological Defense (LMACBD), Military Institute of Engineering (IME), Rio de Janeiro, Brazil; b Brazilian Army Technological Center (CTEx), Institute of CBRN Defense (IDQBRN), Rio de Janeiro, Brazil; c School of Pharmacy, Universidade Castelo Branco (UCB), Rio de Janeiro, Brazil; d Emergency and Rescue Department (DSE), Rio de Janeiro State Fire Department (CBMERJ), Rio de Janeiro, Brazil; e Universidade Estácio de Sá (UNESA), Rio de Janeiro, Brazil; f Instituto Federal de Educação, Ciência e Tecnologia do Rio de Janeiro, Nilópolis, Brazil; g Instituto de Pesquisas de Produtos Naturais Walter Mors (IPPN), Universidade Federal do Rio de Janeiro (UFRJ), Rio de Janeiro, Brazil; h Department of Chemistry, Faculty of Science, University of Hradec Králové, Hradec Králové, Czech Republic

**Keywords:** Isatin, pyridine oximes, organophosphorus poisoning, cholinesterase reactivators, nerve agents, antidotes

## Abstract

Organophosphorus poisoning caused by some pesticides and nerve agents is a life-threating condition that must be swiftly addressed to avoid casualties. Despite the availability of medical countermeasures, the clinically available compounds lack a broad spectrum, are not effective towards all organophosphorus toxins, and have poor pharmacokinetics properties to allow them crossing the blood-brain barrier, hampering cholinesterase reactivation at the central nervous system. In this work, we designed and synthesised novel isatin derivatives, linked to a pyridinium 4-oxime moiety by an alkyl chain with improved calculated properties, and tested their reactivation potency against paraoxon- and NEMP-inhibited acetylcholinesterase in comparison to the standard antidote pralidoxime. Our results showed that these compounds displayed comparable *in vitro* reactivation also pointed by the *in silico* studies, suggesting that they are promising compounds to tackle organophosphorus poisoning.

## 1. Introduction

**Scheme 1.**
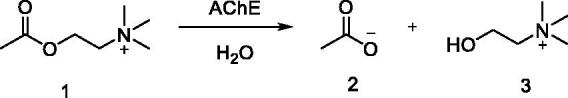
Breakdown of ACh catalysed by AChE.

Acetylcholinesterase (AChE, EC 3.1.1.7) is an enzyme belonging to the family of cholinesterases (ChE), catalysing the hydrolysis of the neurotransmitter acetylcholine (ACh, **1**) in its precursors, acetate (**2**) and choline (**3**) (Scheme 1), and interrupting the nervous impulses in the central and peripheral nervous systems (CNS and PNS)^1–3^. AChE function is of paramount importance, since the accumulation of ACh in the synaptic cleft generates a cholinergic crisis, causing several symptoms such as convulsions, cardiac arrhythmia and, ultimately, death^4^. According to the level of enzyme inhibition, symptoms can be described by the SLUDGEM syndrome (Salivation, Lachrymation, Urination, Defecation, Gastrointestinal disturbs, Emesis, Miosis and Muscular spasms)^5^.

**Figure 1.**
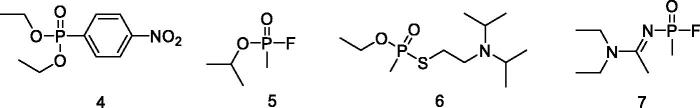
Examples of OP irreversible AChE inhibitors.

In this context, pentavalent organophosphorus compounds (OP) are known irreversible ChE inhibitors, which may interrupt the hydrolysis of ACh by phosphonylation/phosphorylation of the hydroxyl group of a serine residue at the ChE active site. These compounds have been used as pesticides (but some are now prohibited in many countries), and some are listed in the “Annex on Chemicals” of the Chemical Weapons Convention as chemical warfare agents (nerve agents). Examples of these toxic chemicals are paraoxon (PXN, **4**), sarin (**5**), VX (**6**) and A-230 (**7**), which structures are illustrated in Figure 1^6–11^.

**Figure 2.**
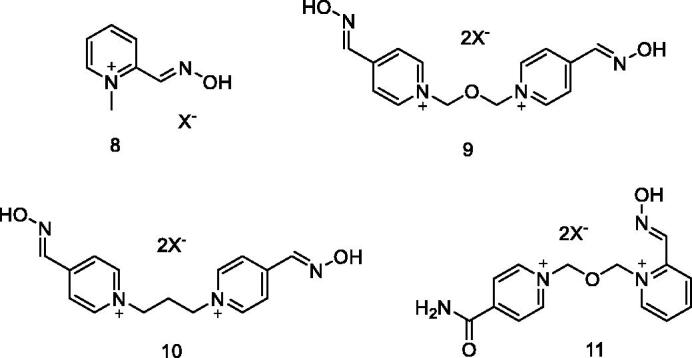
Some current antidotes towards OP poisoning (X^−^ is chloride, bromide or mesylate).

The chemotherapy used in the OP poisoning treatment includes the administration of three types of drugs: an anticholinergic, to antagonise the effects of ACh accumulation; a CNS depressant, which acts as an anticonvulsant, being this optional; and a reactivator of AChE, generally a pyridinium oxime derivative^12^^,^^13^. Despite the use of pyridinium oximes being the current approach to tackle OP poisoning, this therapeutic strategy has a number of disadvantages. Commercial reactivators, like pralidoxime (2-PAM, **8**), obidoxime (**9**), trimedoxime (**10**) and asoxime (**11**) (Figure 2), are effective only against specific inhibitors and have poor permeability into the blood-brain barrier (BBB), rendering reduced enzyme reactivation at the brain, due to their positive charges^14^^,^^15^.

An additional limitation of the current antidotes is their inefficacy to reactivate O-dealkylated OP-AChE adducts (aged enzyme)^16–18^. Thus, the efficacy of the treatment depends on the administration of the antidote immediately upon exposure of the patient to the OP^19^. Therefore, it is not only important to identify the inhibitory agent to be able to employ the most suitable reactivator, but also the further development of novel compounds to address all these situations^20^. Although different methods for OP poisoning treatment have already been proposed, there is still no efficient drug for any type of agent after more than five decades of research^21^.

Recent studies have suggested that monocationic or neutral compounds might be more effective in crossing the BBB^22–26^. Following this line, and encouraged by promising previous results^9^^,^^10^, we designed new isatin derivates linked to the pyridine 4-oxime moiety, yielding monocationic compounds with interesting calculated properties. Taking advantage of the fact that isatins are present in many bioactive compounds, are accessible, and easily modifiable by different reactions^27–30^, we synthesised in the present work, five novel isatin derivatives and further evaluated them as reactivator candidates for two different OP-AChE adducts, paraoxon (PXN) (a pesticide) and NEMP (O-(4-nitrophenyl) O-ethyl methylphosphonate), a VX surrogate, in comparison to pralidoxime (2-PAM, **8**).

## 2. Results

### 2.1. In silico studies

**Figure 3.**
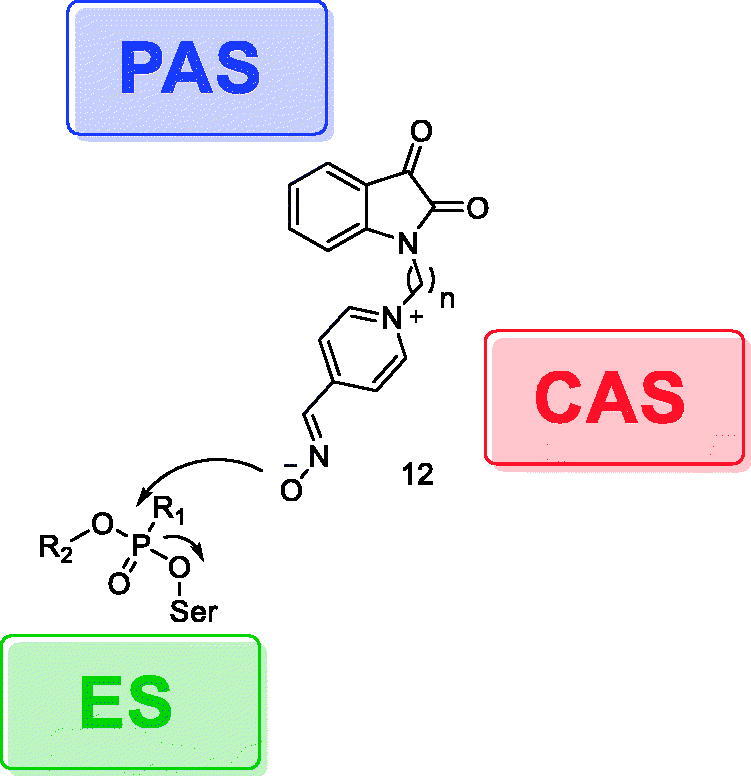
Schematic interactions between the designed compounds and selected AChE sites.

After identifying that isatin-3-oxime^9^^,^^10^ could reactivate at some extent our model of *Electrophorus eel* AChE (*Ee*AChE) inhibited by PXN and NEMP^31–33^, and having the AChE binding sites in mind, we designed a series of compounds bearing isatin as a lipophilic scaffold linked to the active pyridine 4-oxime group by alkyl chains of different lengths (“n” in structure **12** of Figure 3 represents 3–6 methylene units or 3-oxa analogue of pentane-1,5-diyl). We hoped that these designs could not only improve the pharmacokinetics (increasing the calculated logP in comparison to available drugs), but also lead to a better interaction with the important binding sites of AChE. Isatin would then be responsible for interactions with the AChE peripheral anionic site (PAS), which presents mainly aromatic residues, while the linker would increase the lipophilicity, and the pyridinium 4-oxime moiety would be in charge of both the displacement of the phosphorus moiety from the hydroxyl serine residue at the active estearic site (ES), and interactions with the catalytic anionic site (CAS) (Figure 3). By varying the length of the linker, we also could estimate the optimal conformation for achieving the higher reactivation.

Table 1 shows some calculated properties of the designed compounds. LogP was estimated using the Chemicalize server^34^. Results showed higher values than that of 2-PAM, suggesting greater penetration in the BBB. The p*K*a values were also calculated using the Chemicalize server^34^, and values were compatible with that recommended in the literature^35^. “Drug-scores” (DS) were obtained by the OSIRIS Property Explorer^36^, and results were similar to 2-PAM, with compounds **13a**, **13b** and **13e** standing out for having higher values than 2-PAM, suggesting that these isatin derivates might have a lower toxicity.

**Table 1.** Calculated druglikeness of the designed compounds.


Compound	Linker	(X^−^)^a^	p*K*a	logP	DS^b^
**13a**	–(CH_2_)_3_–	Br^−^	8.1	−1.99	0.76
**13b**	–(CH_2_)_4_–	Cl^−^	9.0	−1.54	0.65
**13c**	–(CH_2_)_5_–	Br^−^	9.0	−1.09	0.48
**13d**	–(CH_2_)_6_–	Cl^−^	9.0	−0.63	0.42
**13e**	–(CH_2_)_2_–O–(CH_2_)_2_–	Cl^−^	8.7	−2.54	0.61
2-PAM (**8**)	–	I^-^	7.6	−3.26	0.60

^a^Salts used in experimental assay.

^b^Drug score.

Tables 2 and 3 display data from the docking studies using PXN and VX as human AChE (*Hss*AChE) inhibitors and the designed compounds as reactivators. 2-PAM was also included in this study for comparison. Three hundred poses were obtained for each ligand (compound) and those with the shortest distance between the phosphorus atom of the phosphorylated enzyme and the oxygen atom of the hydroxyimino group were selected for further analysis. The more negative values of energies observed for the ligands suggest that all of them are capable of stabilising better than 2-PAM inside AChE, while the observed P–O distances shorter, or very close to 2-PAM, suggest that they are also capable of getting close enough to the OP to trigger the reactivation reaction. Additionally, for both systems, interactions of all studied ligands (except for **13d** with VX) with the Tyr124 residue were observed, corroborating with literature^37^^,^^38^.

**Table 2.** *In silico* data obtained using PXN as *Hss*AChE inhibitor.

Compound	*E*_inter_^a^ (kcal/mol)	*E*_ligH_^b^ (kcal/mol)	Distance P–O (Å)	H-bonding residues
**13a**	−133.2	−6.2	5.4	Tyr124 (2x), Phe295, Tyr337
**13 b**	−110.9	−7.5	3.5	Tyr124, Phe295, Ser298 (2x)
**13c**	−128.0	−1.8	3.5	Tyr124 (2x), Phe295
**13d**	−134.1	−6.0	4.3	Tyr124 (2x), Phe295
**13e**	−133.8	−8.6	3.7	Tyr124, Phe295, Ser298 (2x)
2−PAM (**8**)	−84.3	−2.5	7.5	Tyr124

^a^Intermolecular interaction energy between ligand and protein.

^b^Total hydrogen bonding energy.

**Table 3.** *In silico* data obtained using VX/NEMP as *Hss*AChE inbibitor.

Compound	*E*_inter_^a^ (kcal/mol)	*E*_ligH_^b^ (kcal/mol)	Distance P–O (Å)	H-bonding residues
**13a**	−138.0	−4.5	5.7	Tyr124, Tyr337 (2x)
**13 b**	−145.2	−3.2	5.2	Tyr337, Trp286
**13c**	−139.4	−4.8	5.7	Tyr124, Tyr337
**13d**	−135.0	−3.5	6.0	Tyr337 (2x)
**13e**	−132.6	−8.3	6.5	Tyr124 (2x), Tyr337
2-PAM (**8**)	−71.2	−4.3	6.3	Tyr124, Tyr337

^a^Intermolecular interaction energy between ligand and protein.

^b^Total hydrogen bonding energy using VX/NEMP as HssAChE inhibitor.

### 2.2. Synthesis of novel compounds

**Scheme 2.**
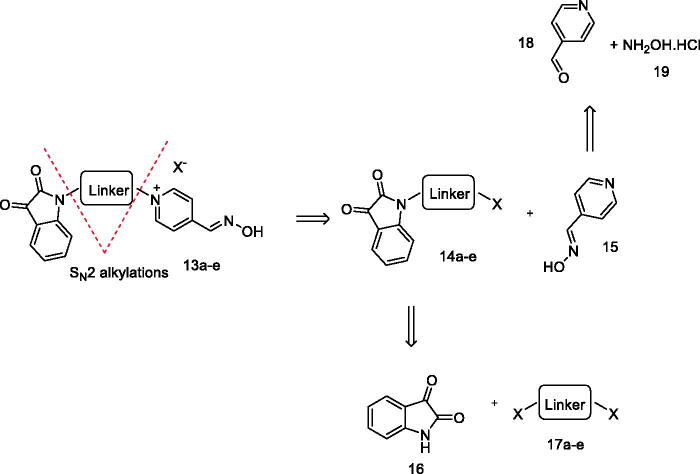
Retrosynthetic analysis for the desired compounds. Linker as defined by Table 1.

The novel isatin-pyridinium oxime hybrids (**13a–e**) were synthesised in a convergent route, as depicted in Scheme 2.

Compounds **13a–e** were synthesised from the reaction between 1,ω-(dihaloalkyl)isatins **14a–e** (1 eq.mol, X is either chlorine or bromine) and the pyridine 4-aldoxime **15** (2 eq.mol) in dry DMF at 80 °C for 96 h (Table 4). Compounds were isolated by trituration (**13 b–e**) and precipitation (**13a**). Yields of bromine-derived oximes were higher than those of chlorine-derived oximes, except for compound **13e**. Compounds **14a–e** were synthesised from isatin (1 eq.mol, **16**) and 1,ω-dihaloalkanes (5 eq.mol, compounds **17a–d**) or bischloroethylether (**17e**) in dry DMF, using anhydrous K_2_CO_3_ (2,5 eq.mol) as base for different times (Table 4). Except for compound **14e**, bromine derivatives showed the shortest reaction times as expected, as it is a better leaving group than chlorine. Pyridine 4-aldoxime **15** was synthesised in accordance with our previous publications^9^^,^^10^.

**Table 4.** Summary of synthesis results.


Linker	15a–e		14a–e	
	Reaction time (h)	Yield (%)	X−	Yield (%)
–(CH_2_)_3_–	3	70	Br	87
–(CH_2_)_4_–	5	81	Cl	62
–(CH_2_)_5_–	3	83	Br	87
–(CH_2_)_6_–	5	73	Cl	22
–(CH_2_)_2_–O–(CH_2_)_2_–	3	77	Cl	25

The *N*-(ω-haloalkyl)isatins **14a–e** were obtained in good yield and purity, despite some difficulties to remove larger linkers, especially 1,6-dichlorohexane (**17d**), although the literature indicates that they are easily removed by chromatographic methods. We tried to reduce the proportion of the linker to three or four equivalents; however, the yield of the desired products was reduced due to the formation of 1,ω-*bis*(indolinyl-2,3-dione)alkanes (“isatin homodimers”), even in short reaction times, or with the addition of nucleophilic catalysts (as KI and tetrabutylammonium bromide – TBAB) or lower temperature (60 or 70 °C). This difficulty to remove excess of 1,6-dichlorohexane was overcome with distillation of its excess before flash chromatography or purification using preparative thin layer chromatography (TLC). This might be one of the reasons for the lower yield of the final product **13e**, which could have reacted with the pyridine 4-aldoxime **15**. We again tried to use nucleophilic catalysts’ approach, but no improvement was achieved so far.

Regarding the purification of **13a–e** compounds, **13a** (1,3-propanediyl linker) could be obtained by simple precipitation using cold acetone. Compounds bearing larger linkers were obtained by trituration with ethyl acetate, exemption given to **13d** (1,6-hexanediyl linker), which was subjected to preparative TLC.

### 2.3. *In vitro* assays

In the studies with AChE inhibited by PXN (Table 5), all compounds presented reactivation^39^, suggesting that isatin derivatives represent a promising strategy for the treatment of intoxication by this OP. All compounds showed maximum reactivation at a concentration greater than or equal to 100 µM, except compound **13d**, whose maximum reactivation occurred at a concentration close to 10 µM. Among the compounds tested, **13c** and **13e** showed the highest percentage of reactivation, with **13c** having the best performance in both concentrations used. Compounds **13b** and **13d**, on the other hand, showed reactivation values higher than 2-PAM only at a concentration of 10 µM. In studies carried out with our model enzyme, *Electrophorus eel* AChE (*Ee*AChE), inhibited by NEMP (Table 5), it was observed that, with the exception of compound **13a**, all showed reactivation^39^, suggesting that isatin derivatives also represent a promising treatment strategy for intoxication by this OP. All compounds showed maximum reactivation at a concentration greater than or equal to 100 µM. Compounds **13c** and **13e** showed the highest reactivation per cent, with values comparable to 2-PAM in both tested concentrations. Compounds **13c** and **13d** also showed activity higher than 10% and about half of that determined for 2-PAM at same concentration. It is noteworthy to inform that the concentration of inhibitors used does not cause full inhibition of enzyme (in our Ellman’s assay, we have been employing concentrations that lead to 85–95% of enzyme activity in comparison to native, non-inhibited enzyme, in the same conditions).

**Table 5.** *In vitro* reactivation results^a^.

	PXN (10 μM)^b^			NEMP (1 μM)^b^		
Compound	1 μM	10 μM	100 μM	1 μM	10 μM	100 μM
**13a**	3 (± 0)	11 (± 0)	15 (± 1)	0 (± 0)	3 (± 1)	3 (± 1)
**13b**	8 (± 2)	37 (± 4)	42 (± 2)	1 (± 1)	2 (± 1)	8 (± 1)
**13c**	15 (± 1)	59 (± 6)	60 (± 3)	1 (± 0)	6 (± 1)	21 (± 1)
**13d**	14 (± 1)	48 (± 5)	32 (± 3)	0 (± 0)	4 (± 0)	6 (± 0)
**13e**	1 (± 0)	14 (± 1)	46 (± 4)	0 (± 0)	5 (± 1)	20 (± 0)
2-PAM (**8**)	33 (± 3)	31 (± 5)	73 (± 5)	0 (± 0)	7 (± 0)	21 (± 0)

^a^Results expressed in percentage of reactivation (mean ± standard deviation).

^b^Final concentration in each well. *Ee*AChE was used as enzyme model and incubated for 30 min with tested inhibitors.

## 3. Discussion

According to Table 1, the calculated p*K*a of isatin derivatives are close to the 2-PAM, suggesting that these derivatives can generate the oximate ion required for the nucleophilic attack on the phosphylated serine residue at physiological conditions. Additionally, they also have higher calculated lipophilicity than the reference compound used in this study, indicating that they might have better permeability into the brain, being able to address the inhibition at the CNS. They also have similar calculated “drug score” that led us to hypothesise that they might have similar profile regarding to toxicity, although further studies are required. As compound **13b** had an estimated toxicity lower than 2-PAM (Table 1), this compound may be an interesting reactivator candidate for PXN-inhibited *Ee*AChE, since it might be administered at concentrations higher than that of conventional oximes.

The designed compounds fared well in *in silico* studies for both inhibitors when compared to 2-PAM. Some of them had the oximate group closer than 2-PAM’s (Tables 2 and 3), suggesting that they can be promising candidates. When *in silico* data were compared to *in vitro* studies, we observed that compounds **13b** and **13d** showed reactivation values higher than 2-PAM at a concentration of 10 µM for PXN-inhibited AChE (Table 5). We also confirmed that the linker length plays an important role for the optimal conformation that led to the highest reactivation rate for this kind of design, as depicted in Figure 3. The fact that compound **13d** (hexane-1,6-diyl linker) displayed higher reactivation at 10 µM in comparison to 100 µM, indicates that this linker length might be an interesting starting point for the development of AChE inhibitors, as the conformation might lead to saturation or conformational changes in the enzymatic sites precluding higher reactivation.

Compounds **13b** and **13c** showed reactivation even at the lowest concentration tested (1 µM), indicating that they may be lead compounds for further development. Both compounds also exhibited similar reactivation rates at 10 and 100 µM in our test conditions. This plateau in the activity might be due to saturation of the enzyme by test compounds, which led us to hypothesise that these compounds may also be reversible inhibitors of *Ee*AChE. In accordance with our preliminary design, we hoped that our compounds could also display such behaviour, making them promising leads for development of prophylactic countermeasures towards OP poisoning and even for neurodegenerative diseases, as Alzheimer’s disease. Therefore, we are also carrying out studies on the inhibitory potential of such derivatives.

Compound **13c** presented the best performance in the *in vitro* assay, for both OP at both reactivator concentrations used (see Table 5), indicating that the linker 1,5-pentanediyl is the best choice to achieve optimal interaction with the AChE binding sites (Figure 3), even at the lowest concentration tested (1 µM). Even exhibiting an estimated drug score relatively lower than 2-PAM (Table 1), reactivation values were similar to 2-PAM, and presented favourable pharmacokinetic properties, such as higher lipophilicity, compared to 2-PAM (Table 1). Based on these results, we speculate that **13c** might be used in lower doses than 2-PAM, making it a promising reactivator for AChE inhibited by PXN and VX.

Compound **13e** showed reactivation values similar to 2-PAM at both concentrations used for AChE inhibited by NEMP (Table 5), and also had an estimated toxicity lower than 2-PAM (see Table 1). Thus, this compound may be administered at concentrations higher than that of conventional oximes, being an interesting reactivator candidate for VX-inhibited AChE.

## 4. Conclusions

Our theoretical and experimental studies showed that the isatin derivatives **13b** and **13e** proved to be promising candidates to address AChE inhibition caused by PXN and NEMP, respectively. Derivative **13c** presented the best *in vitro* performance for both OP at both reactivator concentrations used, being better than 2-PAM for PXN and comparable for VX. Besides, all compounds presented similar reactivation results for PXN. We believe that our results indicate that these novel isatin derivatives are potential scaffolds to be further explored in the design of novel reactivators for OP-inhibited AChE. We are now carrying out studies using the human isoform of AChE to compare results, as well working on improvement of current design to achieve better reactivation rates at the lowest concentrations, proving the potential of the compounds to be prophylactic countermeasures towards such toxicants and analysing the results to find novel valuable AChE inhibitors.

## 5. Materials and methods

### 5.1. General information

All chemicals used in this work were purchased from commercial suppliers and used as received. Isatin, *Ee*AChE (C2888, Type V-S, 1000 U/mg protein), pralidoxime iodide, PXN, 1,ω-dihaloalkanes (1,3-dibromopropane, 1,4-dichlorobutane, 1,5-dibromopentane, 1,6-dichlorohexane, bischloroethyl ether), DMF (dry, sealed bottle) and inorganic compounds (anhydrous potassium carbonate, phosphate salts for buffer solutions, anhydrous sodium sulphate) were purchased from Sigma Aldrich Brazil (São Paulo, Brazil). Acetonitrile and methanol for HPLC-DAD-MS were purchased from Merck Brasil (São Paulo, Brazil). Deuterated solvents (chloroform-d, DMSO-d6, methanol-d_4_, acetone-d_6_) were purchased from Cambridge Isotopes Laboratories (Tewksbury, Massachusetts, USA). Purified water was obtained from Millipore Milli-Q system (18.2 MΩ cm at 25 °C, Millipore Brazil, São Paulo, Brazil). Isolera ACI Chromatography System (Charlotte, NC, USA) was used for flash chromatography. Sealed tubes (Q-Tube) were purchased from Q-Labtech (East Lyme, CT, USA). TLC aluminium plates coated with silica gel F254 were purchased from Merck Brazil (São Paulo, Brazil). Preparative glass TLC coated with silica gel (type KP-Sil, Biotage® HP-Sphere, 25 μ spherical silica, 5 cm × 5 cm) were acquired from Biotage (Charlotte, NC). NMR spectra were obtained from Varian Unity 400 MHz (Santa Clara, CA, USA) and Bruker Avance 400 MHz (Palo Alto, CA, USA) and referred to tetramethylsilane for ^1^H and ^13 ^C NMR spectra. GC-MS (Gas Chromatography-Mass Spectrometry) data were obtained from Agilent 6890 GC system equipped with 5975 C mass spectrometer detector (Billerica, MA, USA). LCMS (liquid chromatography–mass spectrometry) data were obtained from Agilent 1210 LC system equipped with 6410B triple quadrupole mass spectrometer detector (Billerica, MA, USA). SpectraMax Plus 384 microplate reader (Molecular Devices, San Jose, CA, USA) was used in all *in vitro* assays. 96-wells microplates were purchased from Kasvi Brasil (São José dos Pinhais, Paraná, Brazil). Gilson single channel pipettes were purchased from Gilson Inc. (Middleton, WI, USA) and Eppendorf 8-channel pipettes were acquired from Eppendorf Brasil (São Paulo, Brazil). Ellman's tests were performed in triplicate, in three different assays, by at least three different operators, measured at 25 ± 2 °C^40^. Microsoft Excel® 2010 was used for all calculations. All disposable materials and glassware in contact with OP compounds were decontaminated with aqueous solution containing 10% *w/v* NaOH and 10% *w/v* NaClO for 48 h at room temperature in a fume hood before correct destination. Estimations of p*K*a and logP for clinical antidotes and test compounds were obtained from ChemAxon Online Suite^34^. Estimations of DS were obtained from OSIRIS Property Explorer^36^. Agilent Chemstation E.02.02.1431 was used for area integration of GC data for purity calculations. Pyridine-4-aldoxime and NEMP were synthesised in accordance to literature^9^^,^^10^. Full spectroscopic data of all synthesised compounds can be found at the Supplemental material.

### 5.2. General GC-MS and HPLC-MS methods

For GC-MS analysis of *N*-(ω-haloalkyl)isatins **14a–e**, 100 ppm solution of pure compounds in dichloromethane (for reactional mixtures, samples were at same concentration and filtered through a syringe filter 0.22 μm before injection) were prepared. Column: Agilent J&W HP5-MS ((5%-phenyl)-methylpolysiloxane, 30 m × 0.25 mm, 0.25 μ); injection volume, 1 μL; carrier gas: helium; flow rate: 1.8 m–/min; inlet: 170 °C; temperature program: held at 40 °C for 1 min, ramp: 10 °C/min, held at 280 °C for 6 min; MS interface: 250 °C; ionisation energy: 70 eV (electron impact mode).

For LC-MS analysis of isatin-pyridine 4-oxime monocationic hybrids **13a–e**, 10 ppm solution of pure compounds **13a–e** in methanol for reactional mixtures, samples were at same concentration and filtered through a syringe filter 0.22 μm before injection). Column: Agilent Zorbax HILIC (100 × 2.5 × 3.5 μm); injection volume: 1 μL; isocratic elution: 80% acetonitrile (with 0.1% formic acid) and 20% water (with 0.1% formic acid), flow: 0.25 mL/min; ESI-MS mode (positive ion mode); UV at 254 nm (detection: 254 nm/4, reference: 360 nm/100); fragmentator: 70 V; voltage: 4000 V; mass range: *m*/*z* 50–500; gas pressure: 40 psi; vaporiser: 37 °C; gas flow: 10 L/min. MS-MS spectra were acquired in similar conditions to MS spectra: method “Product Ion”; ESI-MS mode (positive ion mode); fragmentator: 70–135 V; collision energy: 20–30 V.

### 5.3. Synthesis of N-(ω-haloalkyl)isatins

In a dry flask (30 min at 130 °C in an oven), under an inert atmosphere and room temperature, isatin **16** (1 mmol) and K_2_CO_3_ (2.5 mmol) were added in dry DMF (4 mL). After 10 min of stirring at room temperature, 1,ω-dihaloalkanes **17a–d** (3 ≤ *ω* ≤ 6) or bischloroethylether **17e** (5 mmol, Table 4) were added. The mixture was heated to 80 °C for different times (see Table 4), and monitored by TLC (silica, using AcOEt/Hex 30:70 as eluent) and GC-MS. After completion of reaction, work-up consisted of addition of AcOEt (40 mL) and successive washes with equal volumes of HCl 1% *v/v* aq and brine (one wash with each solution). The organic phase was separated and dried over anhydrous Na_2_SO_4_. After filtration and concentration of the organic phase, the oily residue was purified by flash chromatography using silica gel (0–40% AcOEt/Hex as eluent). All purified products were orange oils.

### 5.4. Synthesis of isatin-pyridine 4-oxime monocationic hybrids (novel compounds)

*N*-(ω-Haloalkyl)isatins **14a–e** (0.5 mmol) and pyridine 4-aldoxime **15** (1 mmol) were solubilised in ACN (3 mL) in a sealed tube. The reaction solution was maintained at 80 °C for 96 h, being monitored by TLC (50% AcOEt/Hex). After completion of the reaction the solvent was evaporated and products were either isolated by precipitation with cold acetone (20 mL, **13a**, yellow solid) or trituration with ethyl acetate (3 × 3 mL), followed by TLC preparative (AcOEt, **13b–e**, orange oils).

### 5.5. Docking studies

The crystallographic structures of *Hss*AChE complexed with PXN (PDB code: 5HFA) and VX (PDB code: 6CQZ) were used for the docking studies. The 3D structures of each ligand were constructed through the program PC Spartan 08®^41^ and their partial atomic charges calculated through the RM1 (Recife Model 1) semi-empirical method^42^. The software Molegro Virtual Docker (MVD)^®^^43^ was used to perform docking studies through the algorithm MolDock Score, an adaptation of the algorithm Differential Evolution (DE)^44^. Redocking was performed to validate the methodology, using the own inhibitor as reference. The binding site was limited to a sphere with a radius of 15 Å and residues within a 10 Å radius were considered flexible. Due to the stochastic nature of the docking algorithm, about 10 runs were done for each compound, with 30 configurations (poses) returned for evaluation. The best pose of each compound was selected according to the following criteria: distance between the P atom of OP and the O atom of the oxime, interaction energy between the oxime and inhibited AChE, energy involved on hydrogen bonds and total number of AChE residues interacting with the oxime.

### 5.6. Ellman's spectrophotometric assays

The method used is a slight modification of a previous published paper^40^, using 96-wells microplates and a maximum volume of 200 µL. For negative and positive controls, i.e. native enzyme activity and enzyme incubated with inhibitor, respectively, it was pipetted 20 µL of phosphate buffer solution (PBS, pH 7.60 ± 0.10), 70 µL of AChE solution (2.14 U/mL in each well), 80 µL of DTNB 0.4 mg/mL, 10 µL of inhibitor (positive control, inhibition range: 85–95%) or 10 µL of PBS (negative control, A0), and waited for 10 min for inhibition reaction. Then, we added 20 µL of 1 mmol/L of ATCI and read the absorbance to 412 nm in different times. For reactivation studies, it was pipetted 70 µL of *Ee*AChE (2.14 U/mL, prepared from commercial lyophilized), 80 µL of 0.4 mg/mL DTNB, 10 µL of inhibitor and waited for 10 min for inhibition reaction. Following this time, we added 20 µL of standard antidotes or test molecules in different concentrations (100 and 10 µmol/L as final concentrations) and waited for 30 min for reactivation reaction. At last, we added 20 µL of 1 mmol/L of ATCI and read the absorbance (*Ar*) to 412 nm in different times to calculate the enzyme reactivation. AChE inhibition and reactivation rates were given by Equations (1) and (2), respectively. None of the synthesised compounds presented absorbance at assay wavelength nor reacted with test reagents (no oximolysis) at the tested concentrations.

(1) $$\%I=100\times (\frac{A_{0}-A_{i}}{A_{0}})$$

(2) $$\%R=[1-(\frac{A_{0}-A_{r}}{A_{0}-A_{i}})]\times 100$$

## Supplementary Material

Supplemental Material
