# Supplementary material for: Design, synthesis, in silico studies and in vitro evaluation of isatin-pyridine oximes hybrids as novel acetylcholinesterase reactivators
Source: J Enzyme Inhib Med Chem. 2021 Jun 21;36(1):1370–7. doi: 10.1080/14756366.2021.1916009 (PMC8219220; doi:10.1080/14756366.2021.1916009)
Supplement: Supplemental Material [file IENZ_A_1916009_SM8112.pdf]

Design, synthesis, *in silico* studies and *in vitro* evaluation of isatin-pyridine oximes hybrids as novel acetylcholinesterase reactivators.

Daniel A. S. Kitagawa[1,2], Rafael B. Rodrigues[2], Thiago N. Silva[3], Wellington V. dos Santos[4,5], Vinicius C. V. da Rocha[6], Joyce S. F. D. de Almeida[1], Leandro B. Bernardo[2], Taynara Carvalho-Silva[2], Cintia N. Ferreira[2], Angelo A. T. da Silva[6], Alessandro B. C. Simas[7], Eugenie Nepovimova[8], Kamil Kuča[8,], Tanos C. C. França[1,8,*], Samir F. de A. Cavalcante[2,3,7,8,*]*

[1] Laboratory of Molecular Modelling Applied to Chemical and Biological Defense (LMACBD), Military Institute of Engineering (IME), Praça General Tibúrcio 80, Rio de Janeiro 22290-270, Brazil; kitagawa.daniel@ime.eb.br (D.A.S.K.), joycediz@ime.eb.br (J.S.F.D.A.), tanos@ime.eb.br (T.C.C.F.).
[2] Institute of CBRN Defense (IDQBRN), Brazilian Army Technological Center (CTEx); rrodrigues.borges@eb.mil.br (R.B.R.), leandro.braga@eb.mil.br (L.B.B.), taynara.carvalho@eb.mil.br (T.C.-S.), cintia.ferreira@eb.mil.br (C.N.F.), samir.cavalcante@eb.mil.br (S.F.A.C.).
[3] Universidade Castelo Branco (UCB), School of Pharmacy, Avenida de Santa Cruz 1631, Rio de Janeiro 21710-255, Brazil; thnunes92@gmail.com (T.N.S.).
[4] Emergency and Rescue Department (DSE), Rio de Janeiro State Fire Department (CBMERJ), Praça São Salvador 4, Rio de Janeiro 22231-170, Brazil; enfmswv.santos@gmail.com (W.V.S.).
[5] Universidade Estácio de Sá (UNESA), Av. Marechal Fontenele 3555, Jardim Sulacap, Rio de Janeiro, 217401-001, Brazil.
[6] Instituto Federal de Educação, Ciência e Tecnologia do Rio de Janeiro, Nilópolis, 26530-060, Brazil; vcv.darocha@gmail.com (V.C.V.R.), angelo.silva@ifrj.edu.br (A.A.T.S.).
[7] Instituto de Pesquisas de Produtos Naturais Walter Mors (IPPN), Universidade Federal do Rio de Janeiro (UFRJ), CCS, Bloco H, Rio de Janeiro 21941-902, Brazil; abcsimas@nppn.ufrj.br (A.B.C.S.).
[8] Department of Chemistry, Faculty of Science, University of Hradec Králové, Rokitanskeho 62, 50003 Hradec Králové, Czech Republic; Evzenie.N@seznam.cz (E.N.), kamil.kuca@uhk.cz (K.K.).

Supplementary Material – Design, Synthetic Route and Spectral Data of Isatin Derivatives

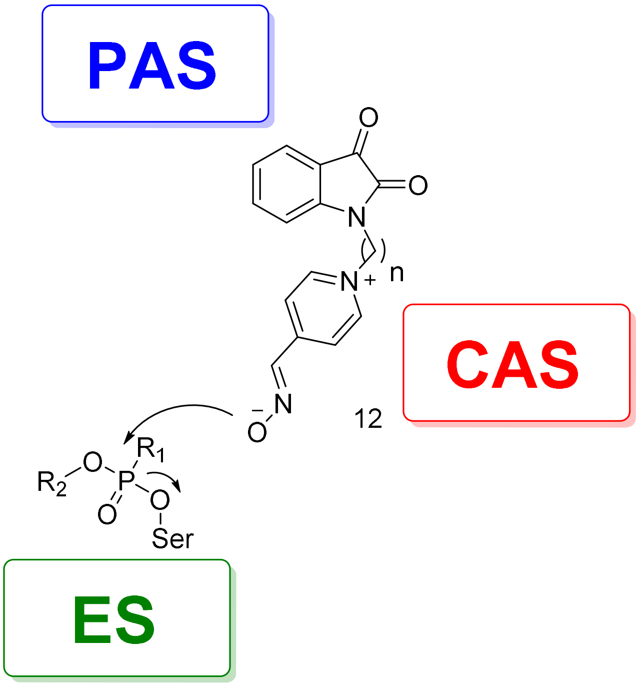

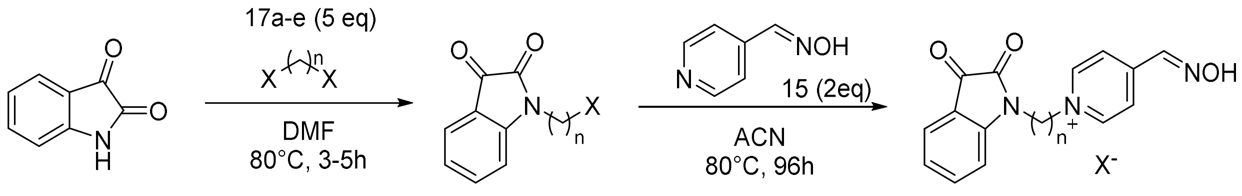

16 (1eq) 14a-e 13a-e

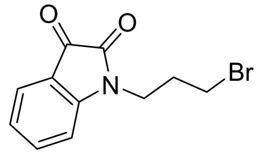

1-(3-bromopropyl)indoline-2,3-dione (14a)

Yield: 70%; ^{1}H-NMR ($CDCl_3$, δ ppm, 500 MHz): 2.29 (q, *J* = 6.43 Hz, 2H), 3.48 (t, *J* = 6.24 Hz, 2H), 3.91 (t, *J* = 7.05 Hz, 2H), 7.03 (m, 1H), 7.14 (td, *J* = 7.58 Hz, 1H), 7.62 (m, 2H). ^{13}C-NMR ($CDCl_3$, δ ppm, 126 MHz): 183.11, 158.38, 150.73, 138.52, 125.64, 123.93, 117.65, 110.08, 38.86, 30.33, 30.03. HPLC-MS-ESI: 268 (^{79}Br, M+1), 270 (^{81}Br, M+1).

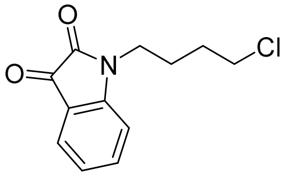

1-(4-chlorobutyl)indoline-2,3-dione (14b)

Yield: 81%; ^{1}H-NMR ($CDCl_3$, δ ppm, 400 MHz): 7.62 (m, 2H), 7.14 (td, *J* = 7.54 Hz, 1H), 6.95 (dt, *J* = 7.81 Hz, 1H), 3.78 (t, *J* = 6.71 Hz, 2H), 3.61 (t, *J* = 5.91 Hz, 2H), 1.90 (t, *J* = 7.29 Hz, 2H). ^{13}C-NMR ($CDCl_3$, δ ppm, 101 MHz): 183.40, 158.21, 150.67, 138.53, 125.51, 123.83, 117.54, 110.18, 44.24, 39.33, 29.46, 24.50. GC-MS-EI: 237 (^{35}Cl, $M^{+\bullet}$), 239 (^{37}Cl, $[M+2]^{+\bullet}$).

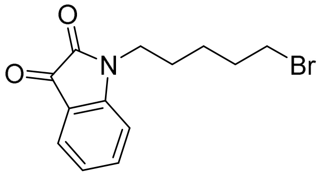

1-(5-bromopentyl)indoline-2,3-dione (14c)

Yield: 83%; ^{1}H-NMR ($CDCl_3$, δ ppm, 400 MHz): 7.61 (m, 2H), 7.14 (td, *J* = 7.57 Hz, 1H), 6.92 (dd, *J* = 8.33 Hz, 1H), 3.75 (t, *J* = 7.25 Hz, 2H), 3.61 (t, *J* = 6.68 Hz, 2H), 1.93 (t, *J* = 14.91 Hz, 2H), 1.74 (m, 2H), 1.57 (m, 2H). ^{13}C-NMR ($CDCl_3$, δ ppm, 101 MHz): 183.49, 158.16, 150.82, 138.44, 125.51, 123.75, 117.54, 110.12, 39.92, 33.32, 32.07, 26.42, 25.37. HPLC-MS-ESI: 295 (^{79}Br, $[M+H]^{+}$), 297 (^{81}Br, $[M+2+H]^{+}$).

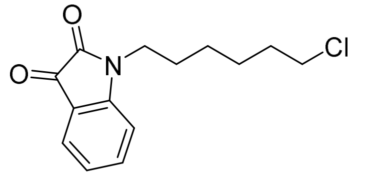

1-(6-chlorohexyl)indoline-2,3-dione (14d)

Yield: 73%; ^{1}H NMR ($CDCl_3$, δ ppm, 500 MHz): 7.60 (m, 2H), 7.12 (dd, *J* = 7.50 Hz, 0.80 Hz, 2H), 6.91 (dd, *J* = 7.90, 0.82 Hz Hz, 2H), 3.73, (t, *J* = 7.25 Hz, 2H), 3.53 (t, *J* = 6.67 Hz, 2H), 1.76 (m, 8H). ^{13}C NMR ($CDCl_3$, δ ppm, 126 MHz): 183.56, 158.21, 150.95, 138.37, 125.51, 123.70, 117.61, 110.13, 44.88, 40.08, 32.35, 27.14, 26.44, 26.18. GC-MS-EI: 265 (^{35}Cl, $M^{+\bullet}$), 267 (^{37}Cl, $[M+2]^{+\bullet}$).

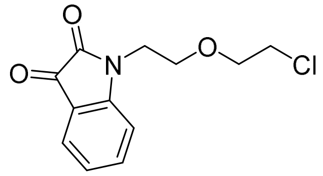

1-(2-(2-chloroethoxy)ethyl)indoline-2,3-dione (14e)

Yield: 77%; ^{1}H-NMR ($CDCl_3$, δ ppm, 400 MHz): 7.60 (m, 2H), 7.11 (m, 2H), 3.95 (t, *J* = 5.18 Hz, 2H), 3.79 (dd, *J* = 5.61 Hz, 4.81 Hz, 2H), 3.72, (dd, *J* = 6.05 Hz, 4.97 Hz, 2H), 3.56 (t, *J* = 5.51 Hz, 2H). ^{13}C-NMR ($CDCl_3$, δ ppm, 101 MHz): 183.35, 158.50, 151.45, 138.33, 125.14, 123.71, 117.48, 111.45, 71.12, 68.85, 42.87, 40.43. HPLC-MS-ESI: 254 (^{35}Cl, $[M+H]^+$), 255 (^{37}Cl, $[M+2+H]^+$).

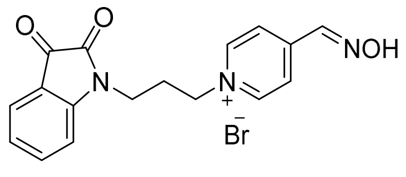

1-(3-(2,3-dioxoindolin-1-yl)propyl)-4-((hydroxyimino)methyl)pyridin-1-ium bromide (13a)

Yield: 87%; ^{1}H-NMR (DMSO-d_6, δ ppm, 400 MHz): 9.00 (d, *J* = 6.4 Hz, 2H), 8.42 (s, 1H), 8.23 (d, *J* = 6.4 Hz, 2H), 7.68 (t, *J* = 7.8 Hz, 1H), 7.60 (d, *J* = 7.4 Hz, 1H), 7.25 (d, *J* = 7.8 Hz, 1H), 7.17 (t, *J* = 7.5 Hz, 1H), 4.66 (t, *J* = 7.7 Hz, 2H), 3.79 (t, *J* = 6.3 Hz, 2H), 2.34 – 2.24 (m, 2H); ^{13}C-NMR (DMSO-d_6, δ ppm, 101 MHz): 183.7, 159.0, 150.7, 148.9, 145.6, 145.5, 138.5, 124.9, 124.5, 123.8, 118.3, 111.1, 58.2, 40.4, 40.4, 40.3, 40.2, 40.1, 40.0, 39.9, 39.9, 39.8, 39.6, 39.4, 36.8, 29.2; HPLC-MS-ESI: 310 (M^+).

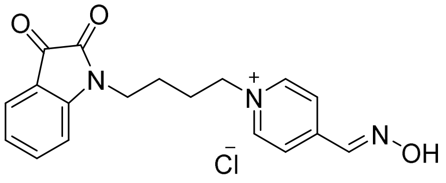

1-(4-(2,3-dioxoindolin-1-yl)butyl)-4-((hydroxyimino)methyl)pyridin-1-ium chloride (13b)

Yield: 62%; ^{1}H-NMR (DMSO-d$_6$, δ ppm, 600 MHz): 9.02 (d, J = 6.9 Hz, 2H), 8.41 (s, 1H), 8.20 (d, J = 6.9 Hz, 2H), 7.65 (td, J = 7.8, 1.4 Hz, 1H), 7.54 (dd, J = 7.4, 1.3 Hz, 1H), 7.19 (d, J = 7.8 Hz, 1H), 7.12 (t, J = 7.5 Hz, 1H), 4.60 (t, J = 7.5 Hz, 2H), 3.71 (t, J = 6.8 Hz, 2H), 1.99 (p, J = 7.6 Hz, 2H), 1.64 (p, J = 6.9 Hz, 2H); ^{13}C-NMR (DMSO-d$_6$, δ ppm, 151 MHz): 186.01, 160.91, 153.06, 151.00, 147.64, 147.53, 140.80, 127.10, 126.64, 125.85, 120.12, 113.32, 62.47, 41.41, 30.46, 25.99; HPLC-MS-ESI: 324 (M$^+$).

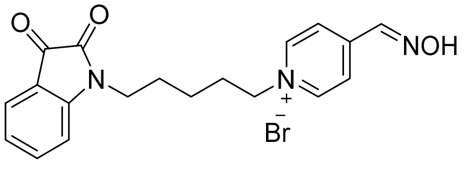

1-(5-(2,3-dioxoindolin-1-yl)pentyl)-4-((hydroxyimino)methyl)pyridin-1-ium bromide (13c)

Yield: 87%; ^{1}H-NMR (DMSO-d$_6$, δ ppm, 600 MHz): 9.01 (d, J = 6.8 Hz, 2H), 8.42 (s, 1H), 8.21 (d, J = 6.9 Hz, 2H), 7.66 (td, J = 7.8, 1.3 Hz, 1H), 7.54 (dd, J = 7.4, 1.3 Hz, 1H), 7.18 (d, J = 7.9 Hz, 1H), 7.13 (t, J = 7.5 Hz, 1H), 4.55 (t, J = 7.5 Hz, 2H), 3.66 (t, J = 7.0 Hz, 2H), 1.94 (m, 2H), 1.65 (p, J = 7.2 Hz, 2H), 1.32 (m, 2H); ^{13}C-NMR (DMSO-d$_6$, δ ppm, 151 MHz): 186.1, 160.7, 153.2, 150.9, 147.7, 147.5, 140.8, 127.1, 126.7, 126.6, 125.8, 120.0, 113.4, 62.8, 42.5, 42.4, 42.2, 42.1, 41.9, 41.8, 41.7, 41.7, 32.7, 28.7, 25.3; HPLC-MS-ESI: 338 (M$^+$).

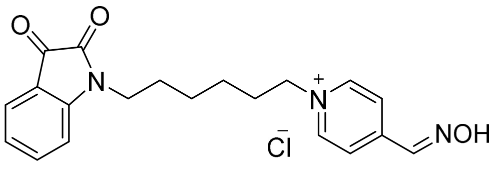

1-(6-(2,3-dioxoindolin-1-yl)hexyl)-4-((hydroxyimino)methyl)pyridin-1-ium chloride (13d)

Yield: 22%; ^{1}H-NMR (DMSO-d$_6$, δ ppm, 600 MHz): 9.03 (d, J = 6.5 Hz, 2H), 8.42 (s, 1H), 8.21 (d, J = 6.9 Hz, 2H), 7.65 (td, J = 7.8, 1.4 Hz, 1H), 7.54 (dd, J = 7.5, 1.3 Hz, 1H), 7.18 (d, J = 7.9 Hz, 1H), 7.12 (t, J = 7.5 Hz, 1H), 4.55 (t, J = 7.3 Hz, 2H), 3.65 (t, J = 7.0 Hz, 2H), 1.89 (p, J = 7.3 Hz, 2H), 1.60 (q, J = 7.2 Hz, 2H), 1.43 – 1.26 (m, 4H); ^{13}C-NMR (DMSO-d$_6$, δ ppm, 151 MHz): 182.3, 156.9, 149.5, 147.2, 143.9, 143.8, 137.1, 123.3, 122.9, 122.0, 116.3, 109.6, 59.1, 38.8, 38.6, 38.5, 38.4, 38.2, 38.1, 38.1, 37.9, 29.3, 25.3, 24.4, 23.8; HPLC-MS-ESI: 352 (M$^+$).

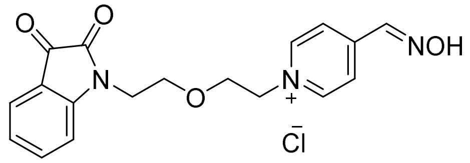

1-(2-(2-(2,3-dioxoindolin-1-yl)ethoxy)ethyl)-4-((hydroxyimino)methyl)pyridin-1-ium chloride (13e)

Yield: 25%; ^{1}H-NMR (DMSO-d_6, δ ppm, 600 MHz): 8.86 (d, J = 6.6 Hz, 2H), 8.38 (s, 1H), 8.02 (d, J = 6.6 Hz, 2H), 7.58 (td, J = 7.8, 1.2 Hz, 1H), 7.50 (dd, J = 7.5, 1.0 Hz, 1H), 7.09 (t, J = 7.5 Hz, 1H), 7.07 (d, J = 7.9 Hz, 1H), 4.72 (t, J = 4.9 Hz, 2H), 3.91 (t, J = 4.9 Hz, 2H), 3.81 (t, J = 5.1 Hz, 2H), 3.69 (t, J = 5.1 Hz, 2H); ^{13}C-NMR (DMSO-d_6, δ ppm, 151 MHz): 185.8, 160.7, 153.3, 151.1, 147.8, 147.5, 140.8, 127.0, 126.0, 125.7, 119.8, 113.6, 70.7, 69.9, 62.3, 42.5, 42.4, 42.2, 42.1, 42.0, 41.9, 41.8, 41.7; HPLC-MS-ESI: 340 (M^+).

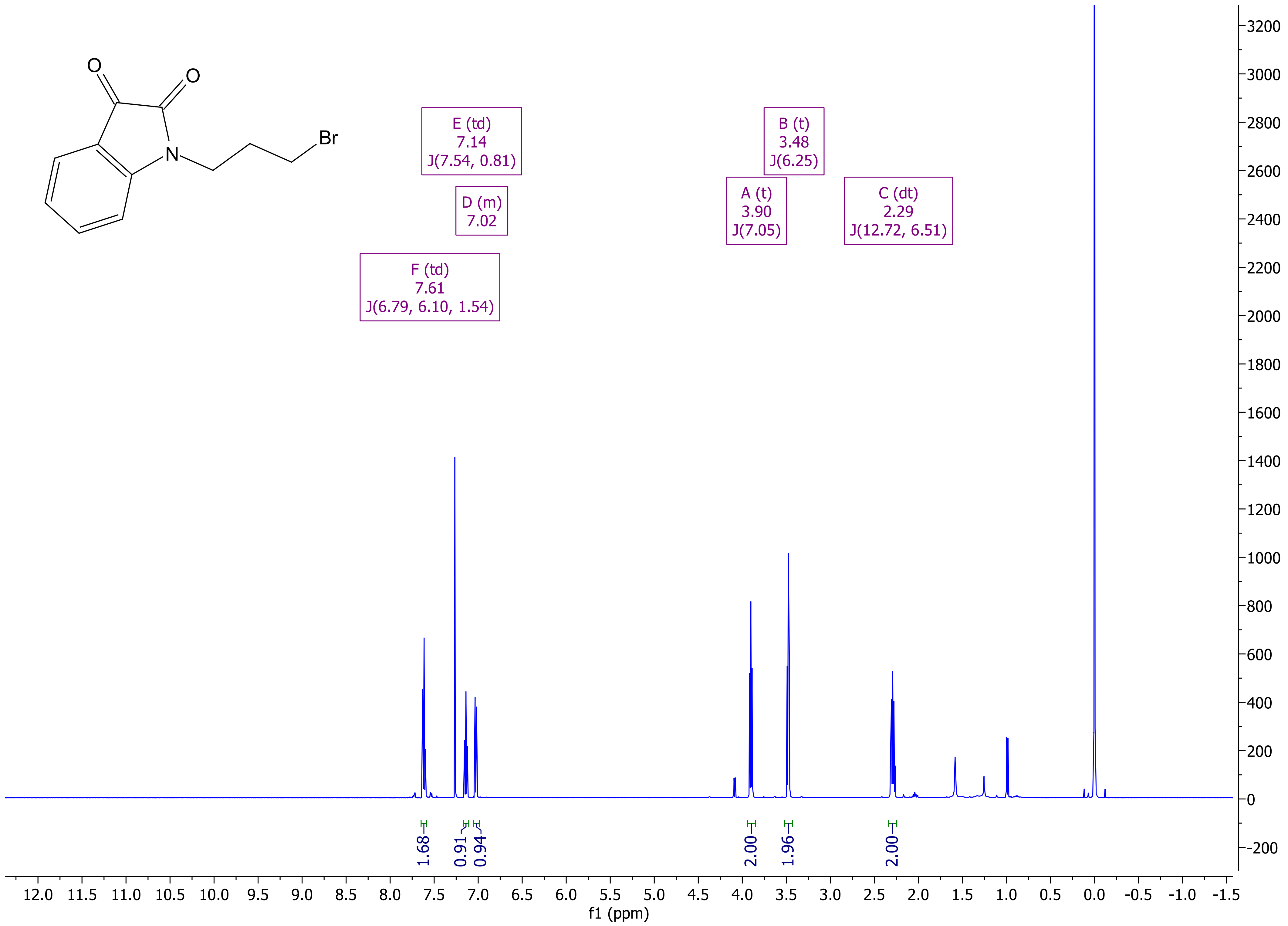
O
O
N
Br
E (td)
7.14
J(7.54, 0.81)
B (t)
3.48
J(6.25)
D (m)
7.02
A (t)
3.90
J(7.05)
C (dt)
2.29
J(12.72, 6.51)
F (td)
7.61
J(6.79, 6.10, 1.54)
1.68
0.91
0.94
2.00
1.96
2.00
12.0 11.5 11.0 10.5 10.0 9.5 9.0 8.5 8.0 7.5 7.0 6.5 6.0 5.5 5.0 4.5 4.0 3.5 3.0 2.5 2.0 1.5 1.0 0.5 0.0 -0.5 -1.0 -1.5
f1 (ppm)
3200 3000 2800 2600 2400 2200 2000 1800 1600 1400 1200 1000 800 600 400 200 0 -200

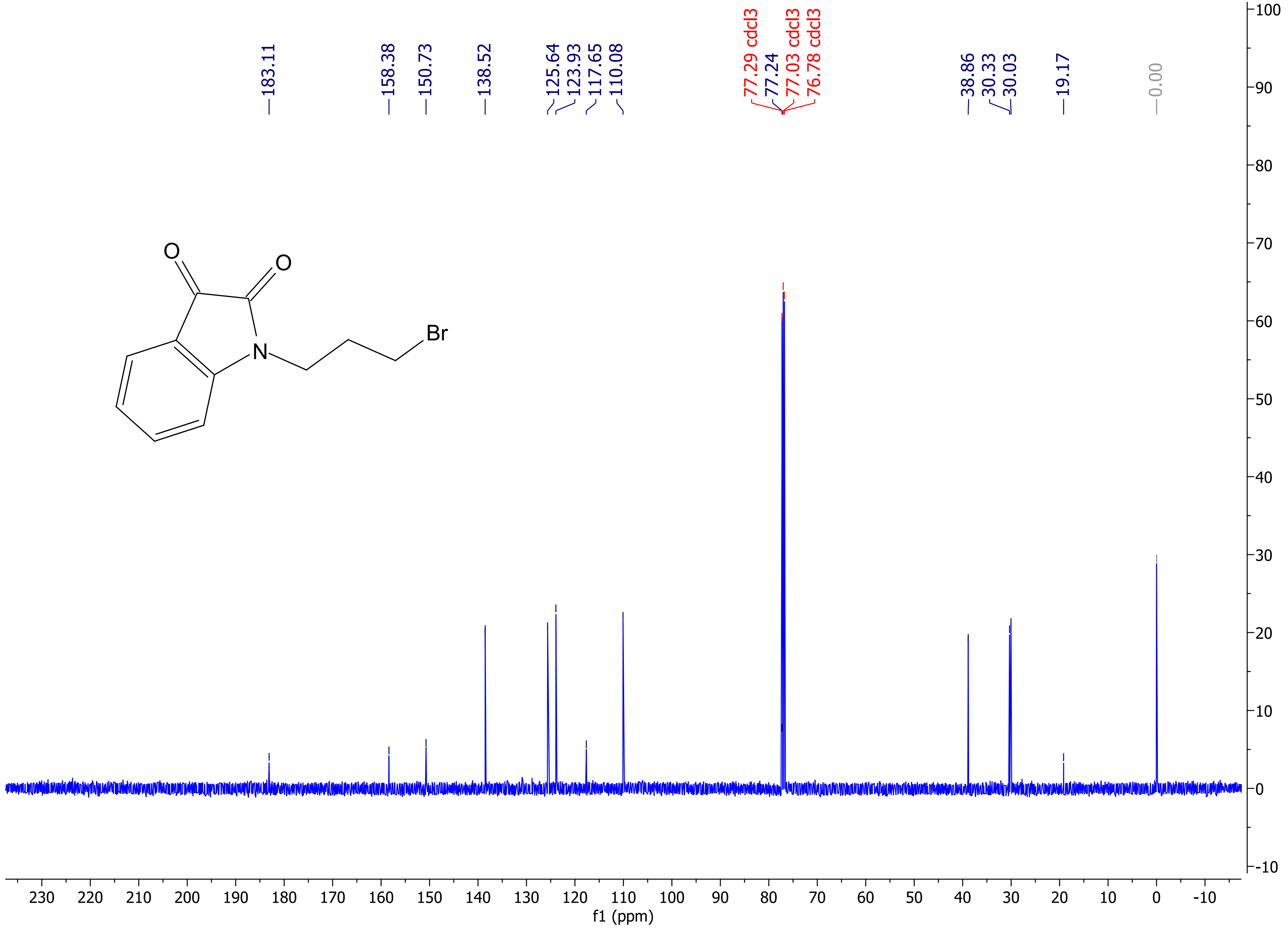
183.11
158.38
150.73
138.52
125.64
123.93
117.65
110.08
77.29 cdcl3
77.24
77.03 cdcl3
76.78 cdcl3
38.86
30.33
30.03
19.17
0.00
O
O
N
Br
f1 (ppm)

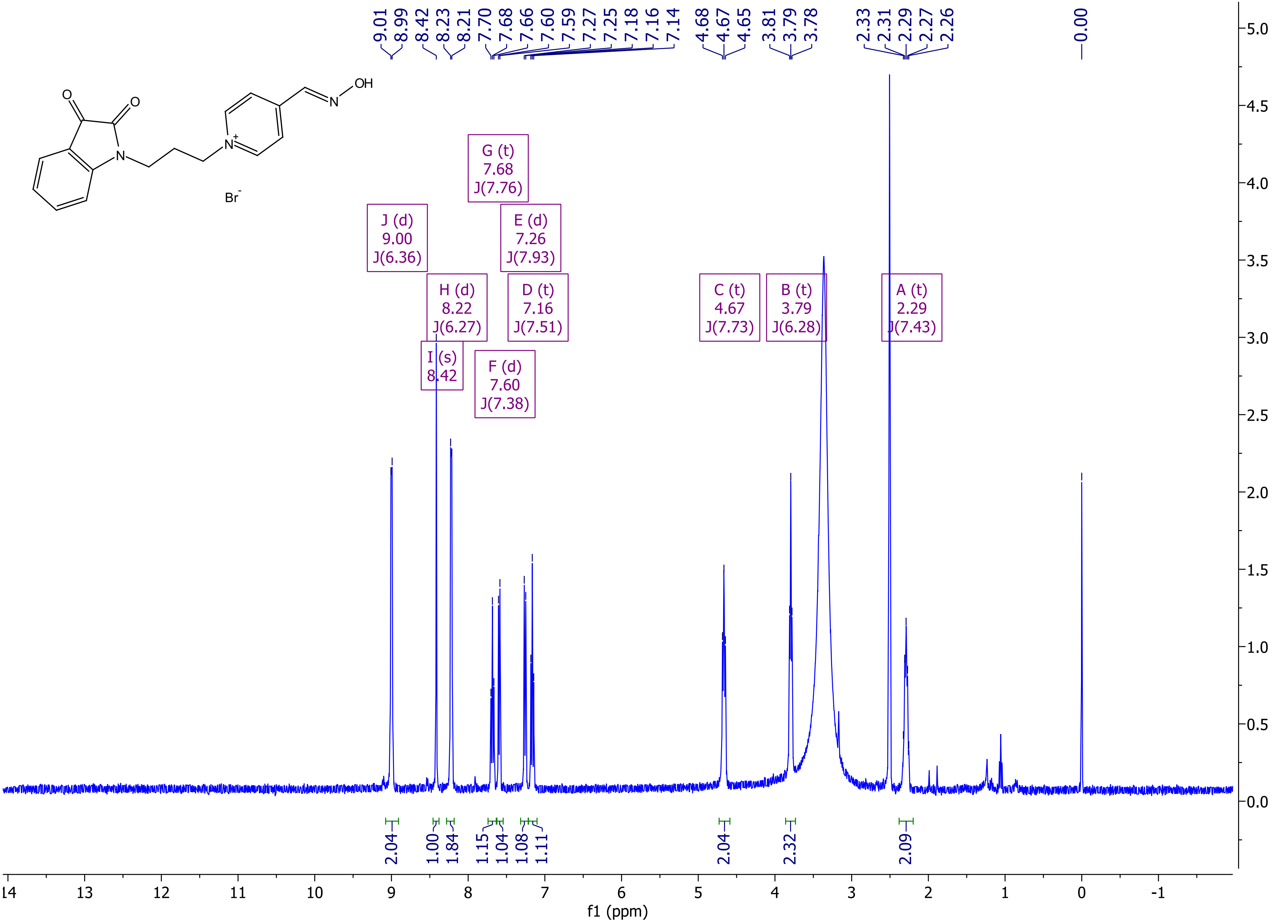
O
O
N
N+
OH
N
Br-
9.01 8.99 8.42 8.23 8.21 7.70 7.68 7.66 7.60 7.59 7.27 7.25 7.18 7.16 7.14 4.68 4.67 4.65 3.81 3.79 3.78 2.33 2.31 2.29 2.27 2.26 0.00
G (t) 7.68 J(7.76)
J (d) 9.00 J(6.36)
E (d) 7.26 J(7.93)
H (d) 8.22 J(6.27)
D (t) 7.16 J(7.51)
I (s) 8.42
F (d) 7.60 J(7.38)
C (t) 4.67 J(7.73)
B (t) 3.79 J(6.28)
A (t) 2.29 J(7.43)
2.04 1.00 1.84 1.15 1.04 1.08 1.11 2.04 2.32 2.09
f1 (ppm)

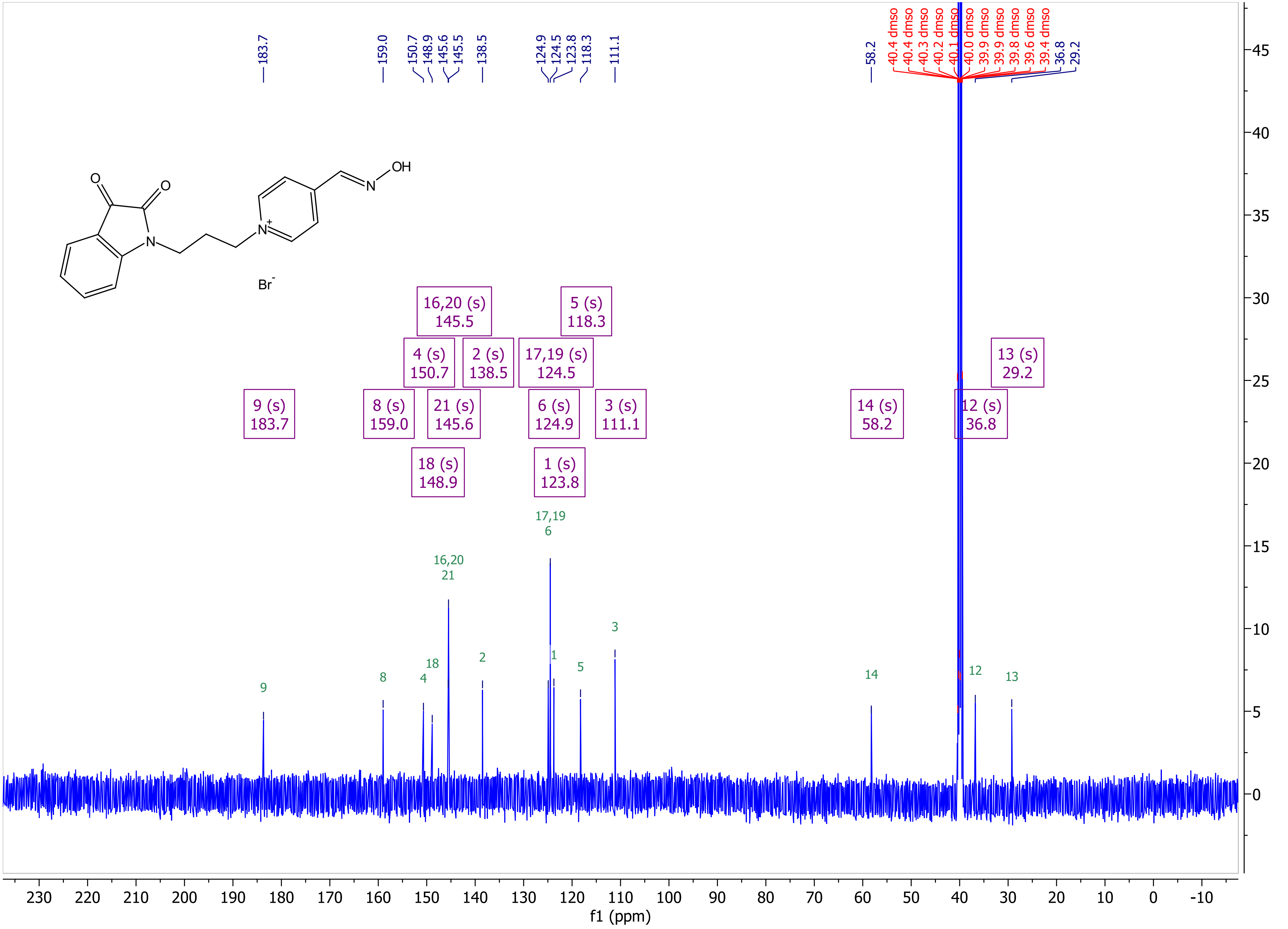

183.7
159.0
150.7
148.9
145.6
145.5
138.5
124.9
124.5
123.8
118.3
111.1
58.2
40.4 dmso
40.4 dmso
40.3 dmso
40.2 dmso
40.1 dmso
40.0 dmso
39.9 dmso
39.9 dmso
39.8 dmso
39.6 dmso
39.4 dmso
36.8
29.2
O
O
OH
N
N
N+
Br-
16,20 (s) 145.5
5 (s) 118.3
4 (s) 150.7
2 (s) 138.5
17,19 (s) 124.5
13 (s) 29.2
9 (s) 183.7
8 (s) 159.0
21 (s) 145.6
6 (s) 124.9
3 (s) 111.1
14 (s) 58.2
12 (s) 36.8
18 (s) 148.9
1 (s) 123.8
17,19
6
16,20
21
3
18
2
1
5
8
4
9
14
12
13
230 220 210 200 190 180 170 160 150 140 130 120 110 100 90 80 70 60 50 40 30 20 10 0 -10
f1 (ppm)
45 40 35 30 25 20 15 10 5 0

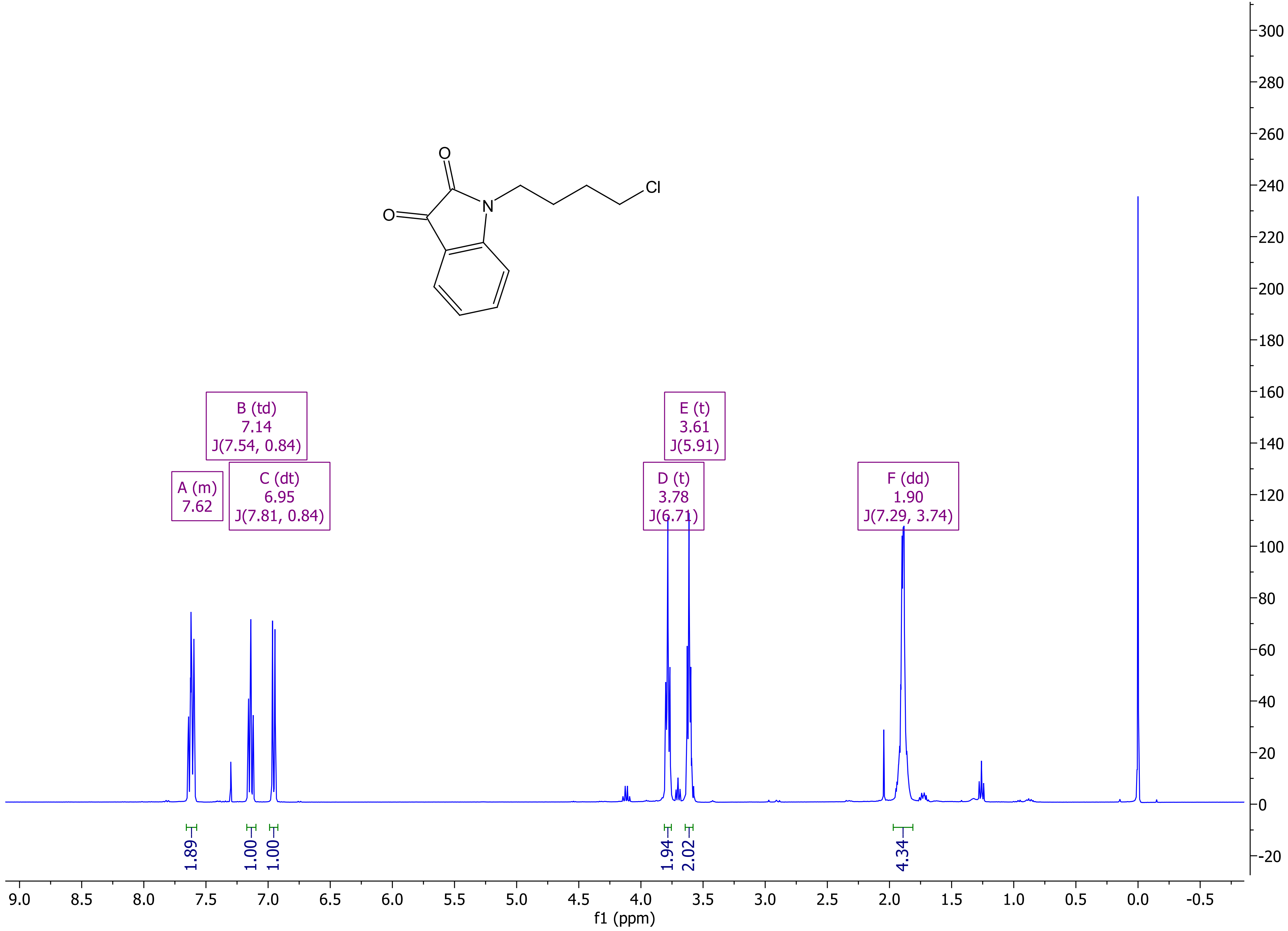
O
O
N
Cl
A (m)
7.62
B (td)
7.14
J(7.54, 0.84)
C (dt)
6.95
J(7.81, 0.84)
D (t)
3.78
J(6.71)
E (t)
3.61
J(5.91)
F (dd)
1.90
J(7.29, 3.74)
1.89
1.00
1.00
1.94
2.02
4.34
9.0
8.5
8.0
7.5
7.0
6.5
6.0
5.5
5.0
4.5
4.0
3.5
3.0
2.5
2.0
1.5
1.0
0.5
0.0
-0.5
f1 (ppm)
300
280
260
240
220
200
180
160
140
120
100
80
60
40
20
0
-20

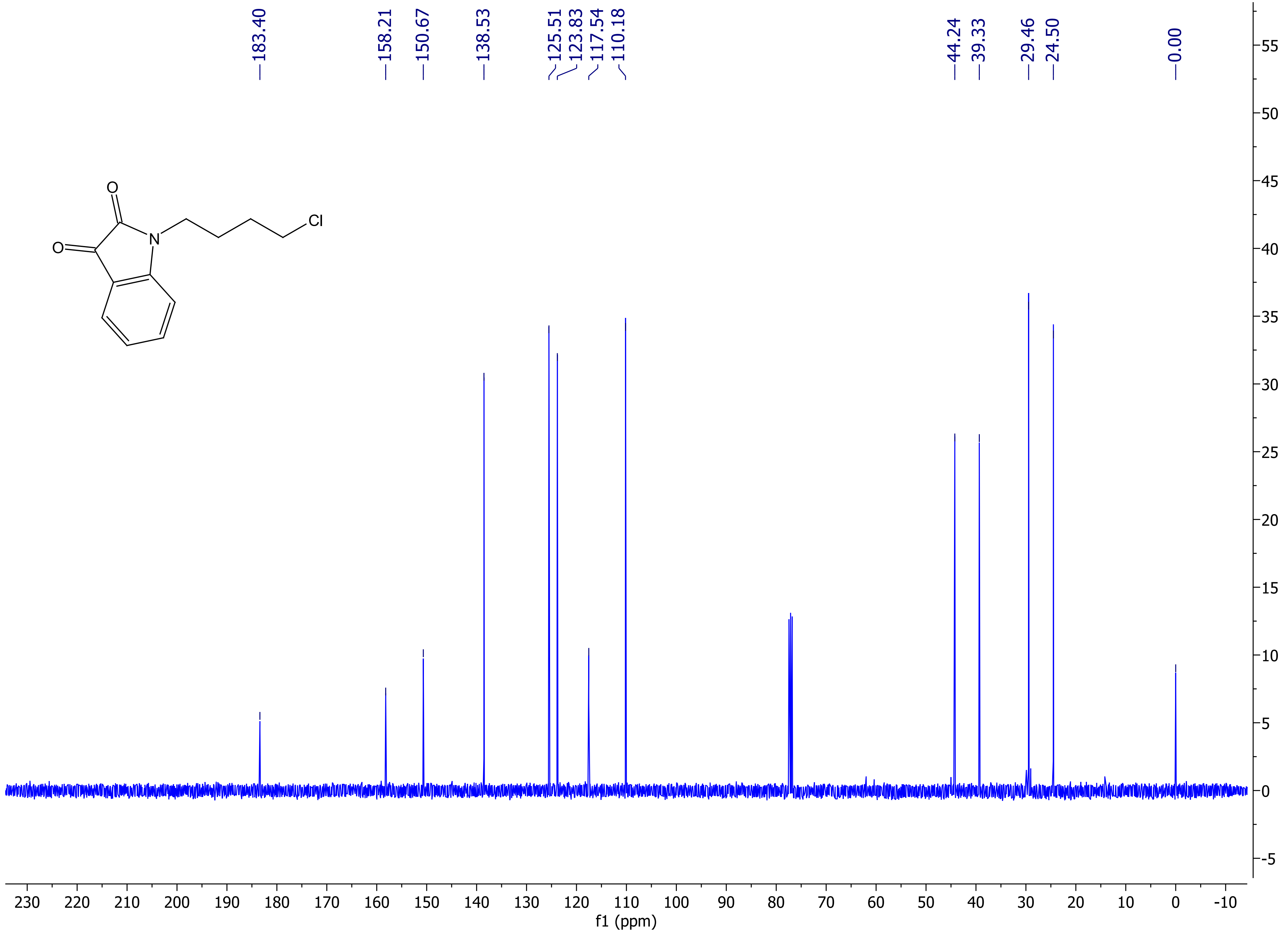

183.40
158.21
150.67
138.53
125.51
123.83
117.54
110.18
44.24
39.33
29.46
24.50
0.00
O
O
N
Cl
f1 (ppm)

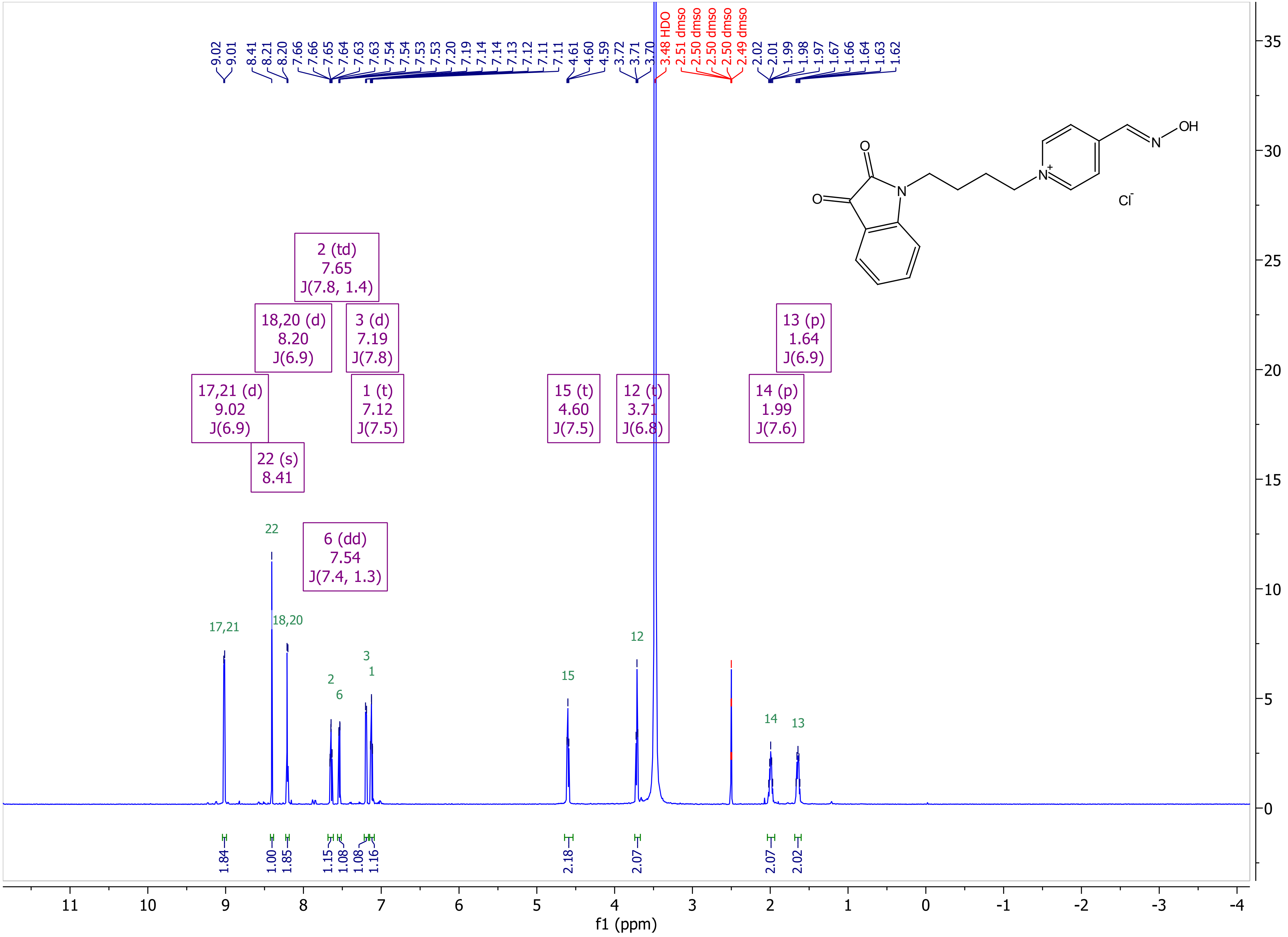

9.02 9.01 8.41 8.21 8.20 7.66 7.66 7.65 7.64 7.63 7.63 7.54 7.54 7.53 7.53 7.20 7.19 7.14 7.14 7.13 7.12 7.11 7.11 4.61 4.60 4.59 3.72 3.71 3.70 3.48 HDO 2.51 dmso 2.50 dmso 2.50 dmso 2.50 dmso 2.49 dmso 2.02 2.01 1.99 1.98 1.97 1.67 1.66 1.64 1.63 1.62
2 (td) 7.65 J(7.8, 1.4)
18,20 (d) 8.20 J(6.9)
3 (d) 7.19 J(7.8)
13 (p) 1.64 J(6.9)
17,21 (d) 9.02 J(6.9)
1 (t) 7.12 J(7.5)
15 (t) 4.60 J(7.5)
12 (t) 3.71 J(6.8)
14 (p) 1.99 J(7.6)
22 (s) 8.41
6 (dd) 7.54 J(7.4, 1.3)
O O N N+ N OH Cl-
1.84 1.00 1.85 1.15 1.08 1.08 1.16 2.18 2.07 2.07 2.02
f1 (ppm)

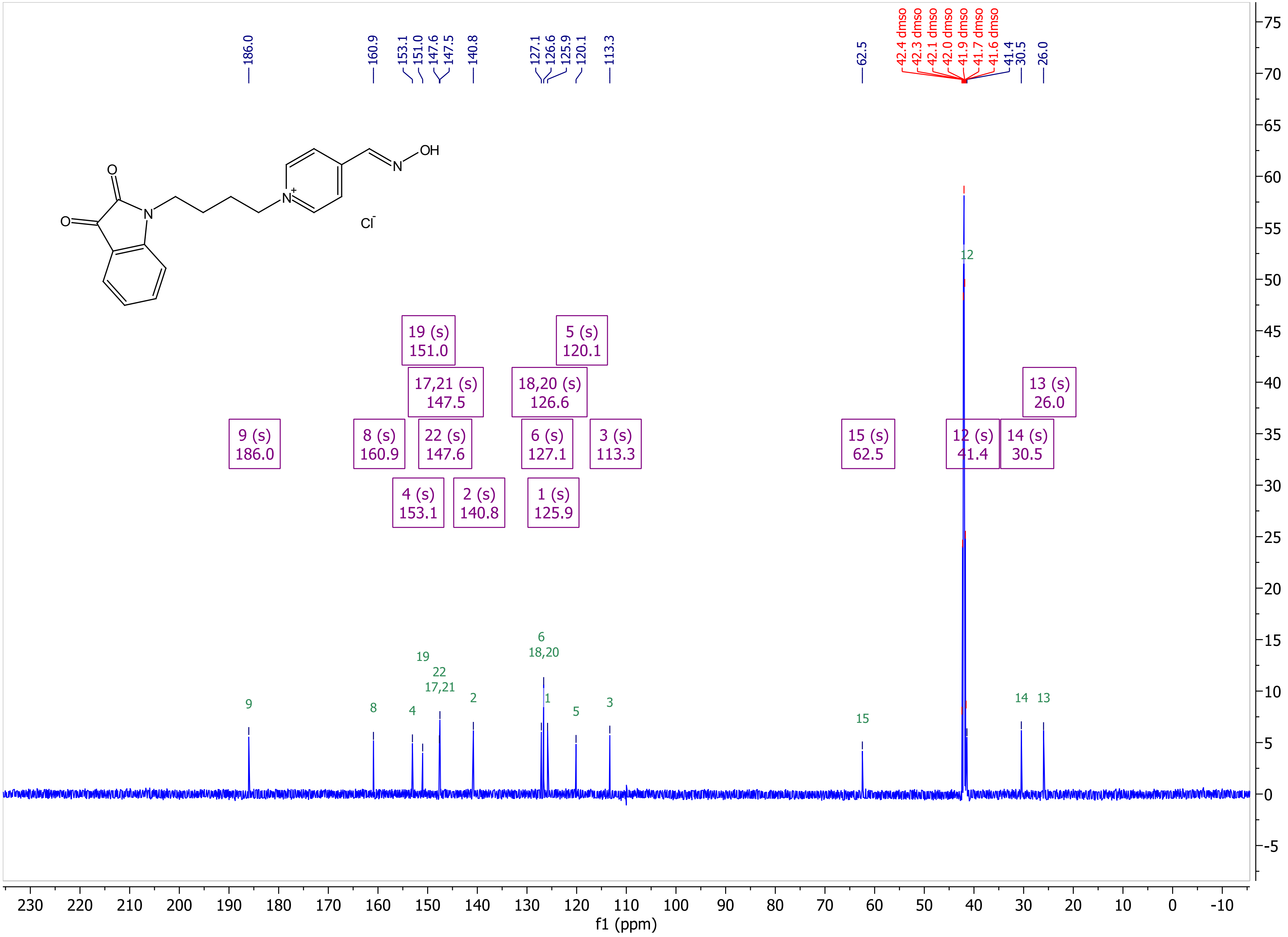

186.0
160.9
153.1
151.0
147.6
147.5
140.8
127.1
126.6
125.9
120.1
113.3
62.5
42.4 dmso
42.3 dmso
42.1 dmso
42.0 dmso
41.9 dmso
41.7 dmso
41.6 dmso
41.4
30.5
26.0
O
O
N
N+
OH
N
Cl-
19 (s) 151.0
5 (s) 120.1
17,21 (s) 147.5
18,20 (s) 126.6
13 (s) 26.0
9 (s) 186.0
8 (s) 160.9
22 (s) 147.6
6 (s) 127.1
3 (s) 113.3
15 (s) 62.5
12 (s) 41.4
14 (s) 30.5
4 (s) 153.1
2 (s) 140.8
1 (s) 125.9
f1 (ppm)

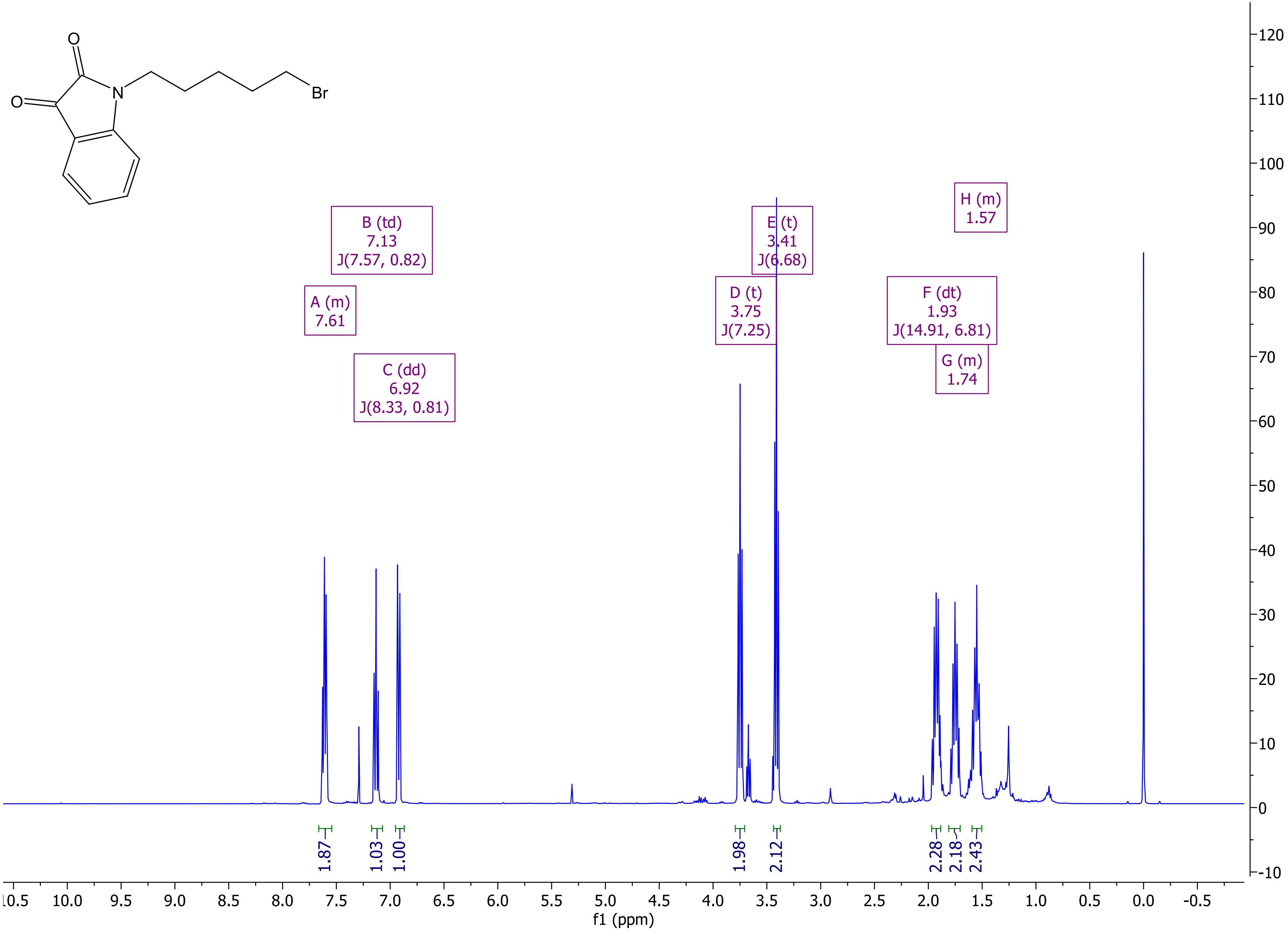

O
O
N
Br
B (td)
7.13
J(7.57, 0.82)
A (m)
7.61
C (dd)
6.92
J(8.33, 0.81)
E (t)
3.41
J(6.68)
D (t)
3.75
J(7.25)
H (m)
1.57
F (dt)
1.93
J(14.91, 6.81)
G (m)
1.74
1.87
1.03
1.00
1.98
2.12
2.28
2.18
2.43
f1 (ppm)

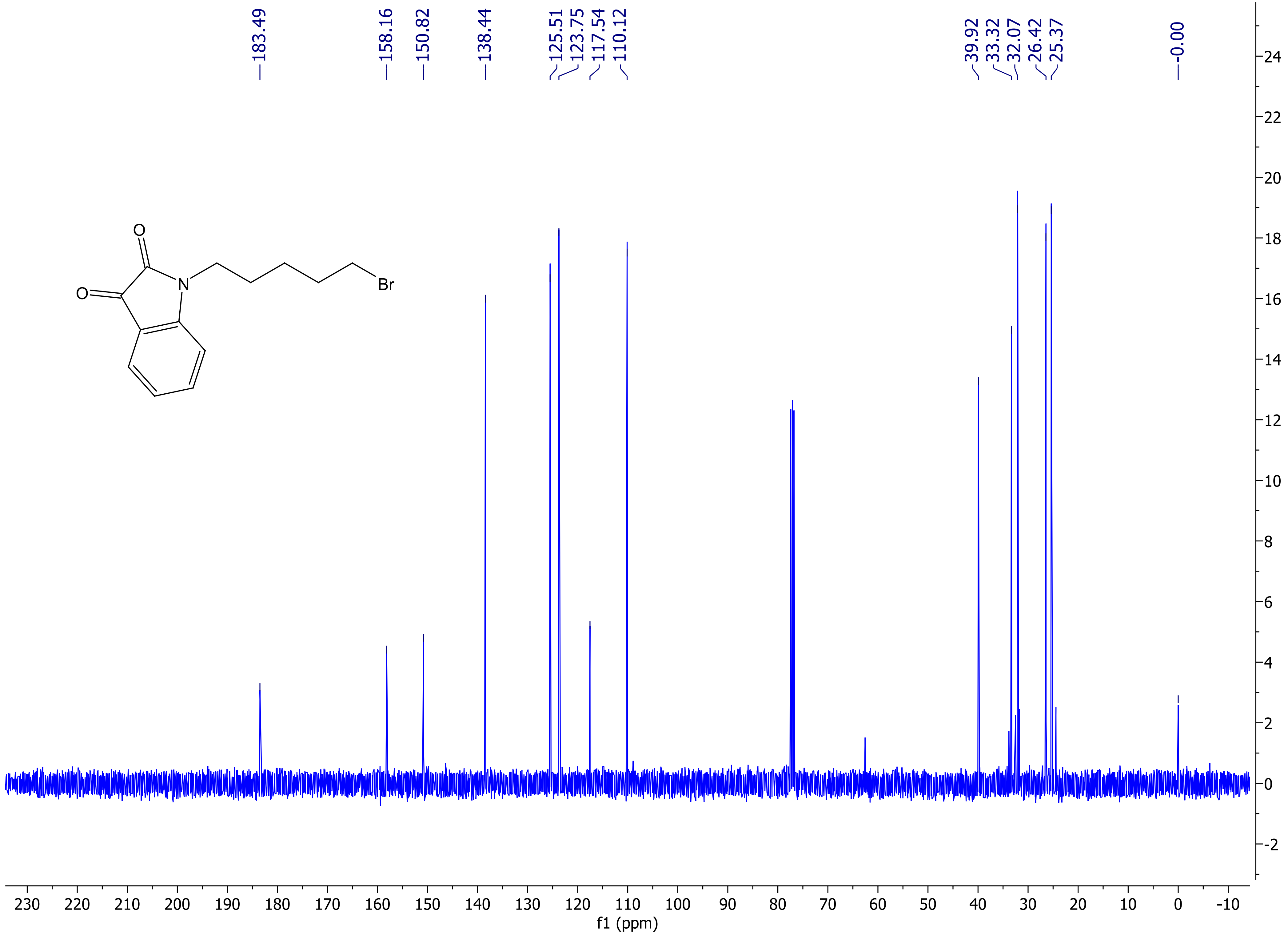

183.49
158.16
150.82
138.44
125.51
123.75
117.54
110.12
39.92
33.32
32.07
26.42
25.37
-0.00
O
O
N
Br
f1 (ppm)

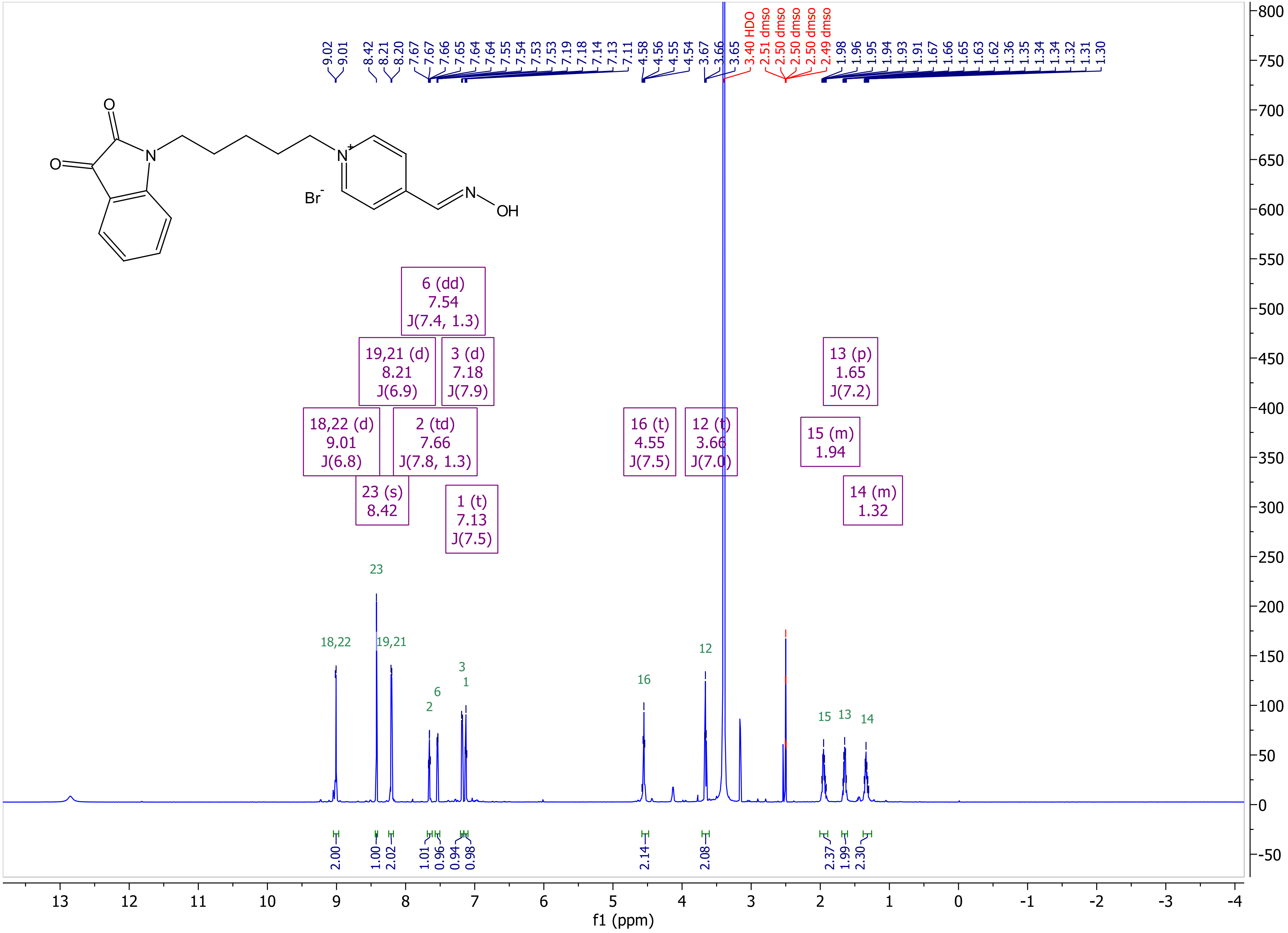

9.02
9.01
8.42
8.21
8.20
7.67
7.67
7.66
7.65
7.64
7.64
7.55
7.54
7.53
7.53
7.19
7.18
7.14
7.13
7.11
4.58
4.56
4.55
4.54
3.67
3.66
3.65
3.40 HDO
2.51 dmso
2.50 dmso
2.50 dmso
2.50 dmso
2.49 dmso
1.98
1.96
1.95
1.94
1.93
1.91
1.67
1.66
1.65
1.63
1.62
1.36
1.35
1.34
1.34
1.32
1.31
1.30
O
O
N
N+
Br-
N
OH
6 (dd) 7.54 J(7.4, 1.3)
19,21 (d) 8.21 J(6.9)
3 (d) 7.18 J(7.9)
13 (p) 1.65 J(7.2)
18,22 (d) 9.01 J(6.8)
2 (td) 7.66 J(7.8, 1.3)
16 (t) 4.55 J(7.5)
12 (t) 3.66 J(7.0)
15 (m) 1.94
14 (m) 1.32
23 (s) 8.42
1 (t) 7.13 J(7.5)
23
18,22
19,21
3
1
6
2
16
12
15
13
14
2.00
1.00
2.02
1.01
0.96
0.94
0.98
2.14
2.08
2.37
1.99
2.30
f1 (ppm)

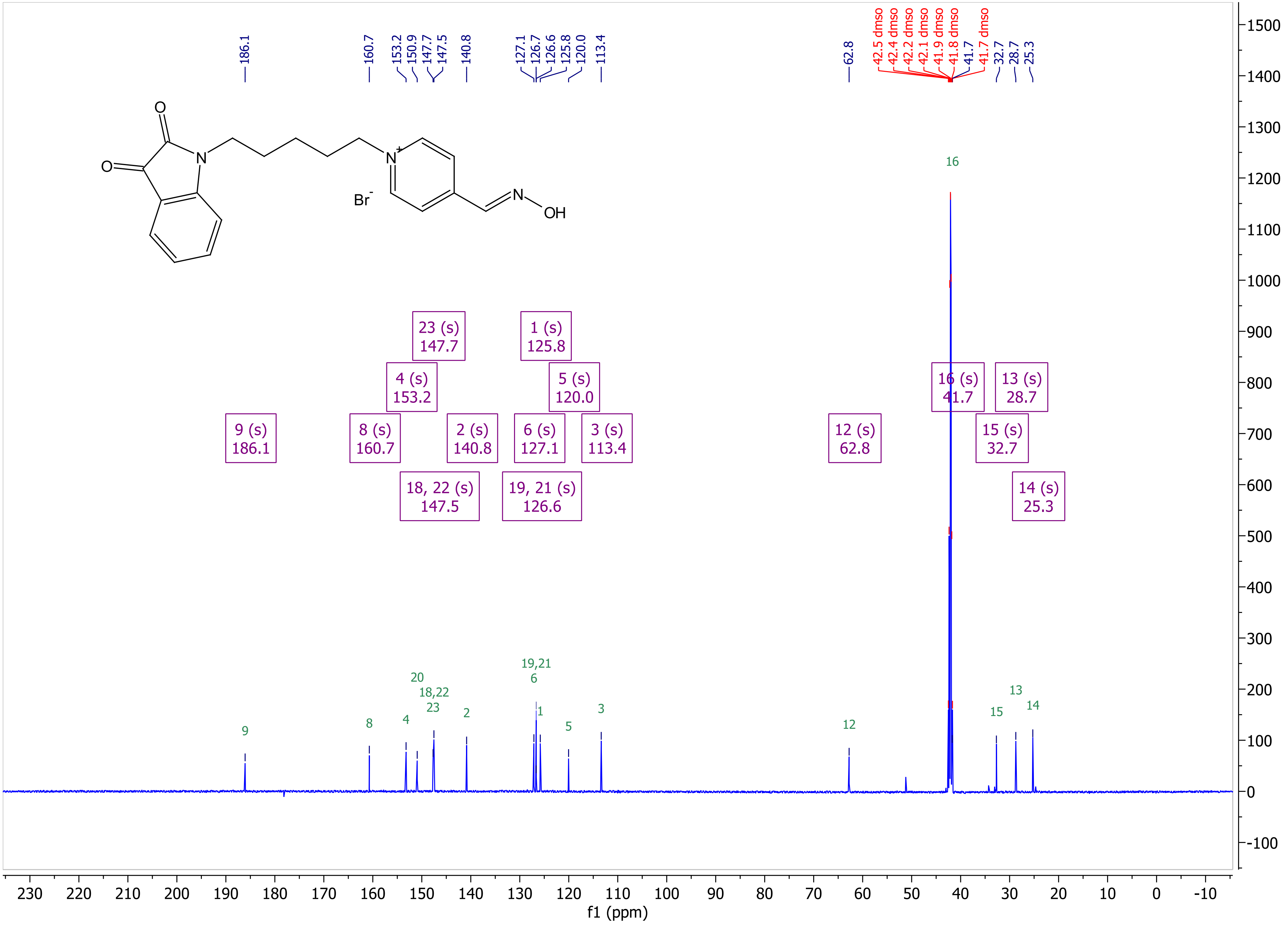

186.1
160.7
153.2
150.9
147.7
147.5
140.8
127.1
126.7
126.6
125.8
120.0
113.4
62.8
42.5 dmso
42.4 dmso
42.2 dmso
42.1 dmso
41.9 dmso
41.8 dmso
41.7
41.7 dmso
32.7
28.7
25.3
O
O
N
N+
Br-
N
OH
23 (s) 147.7
1 (s) 125.8
4 (s) 153.2
5 (s) 120.0
16 (s) 41.7
13 (s) 28.7
9 (s) 186.1
8 (s) 160.7
2 (s) 140.8
6 (s) 127.1
3 (s) 113.4
12 (s) 62.8
15 (s) 32.7
18, 22 (s) 147.5
19, 21 (s) 126.6
14 (s) 25.3
16
9
8
4
20
18,22
23
2
19,21
6
1
5
3
12
15
13
14
f1 (ppm)

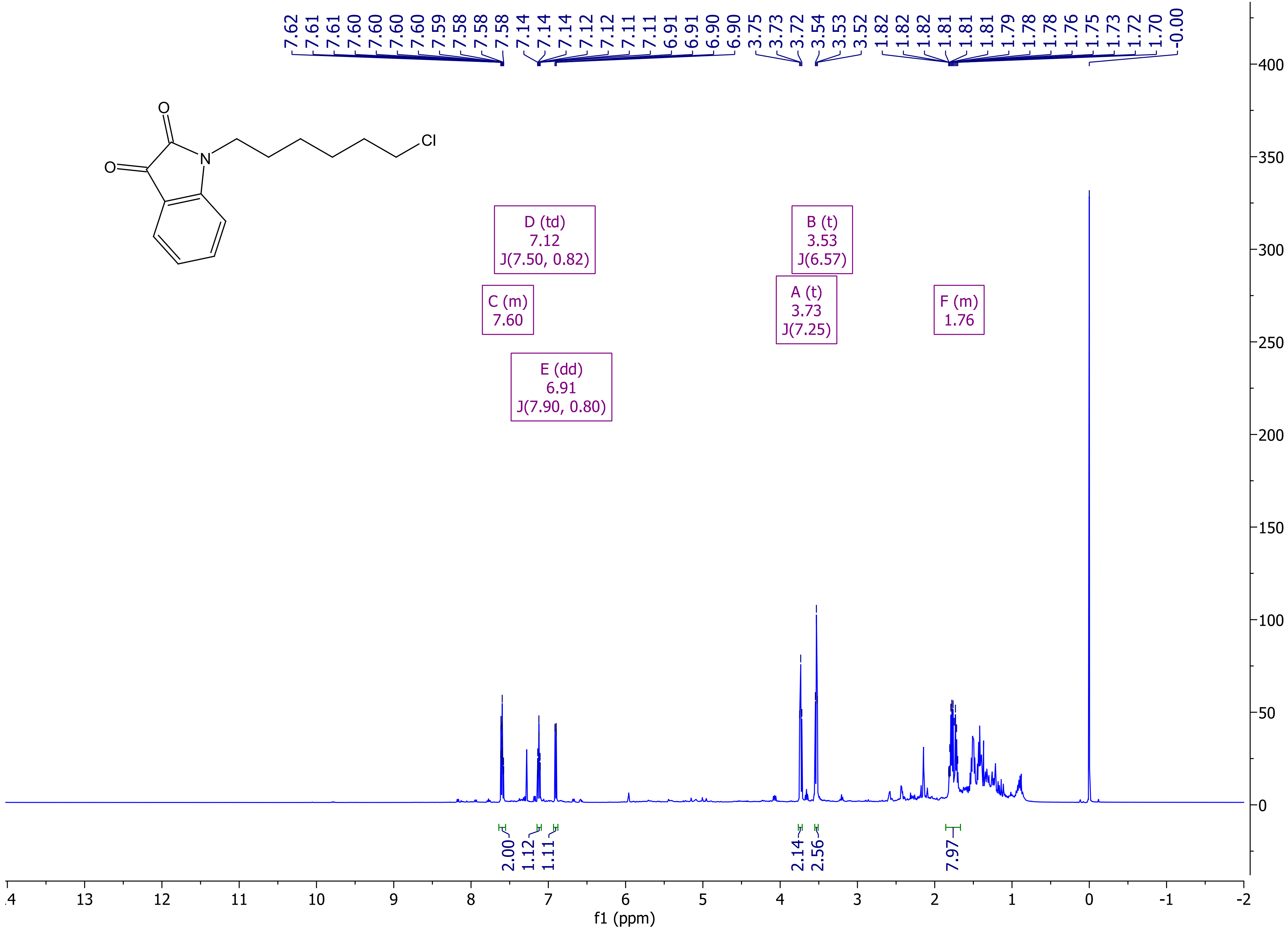

7.62 7.61 7.61 7.60 7.60 7.60 7.60 7.59 7.58 7.58 7.58 7.14 7.14 7.14 7.12 7.12 7.11 7.11 6.91 6.91 6.90 6.90 3.75 3.73 3.72 3.54 3.53 3.52 1.82 1.82 1.82 1.81 1.81 1.81 1.79 1.78 1.78 1.76 1.75 1.73 1.72 1.70 -0.00
O
O
N
Cl
D (td)
7.12
J(7.50, 0.82)
C (m)
7.60
E (dd)
6.91
J(7.90, 0.80)
B (t)
3.53
J(6.57)
A (t)
3.73
J(7.25)
F (m)
1.76
2.00
1.12
1.11
2.14
2.56
7.97
f1 (ppm)

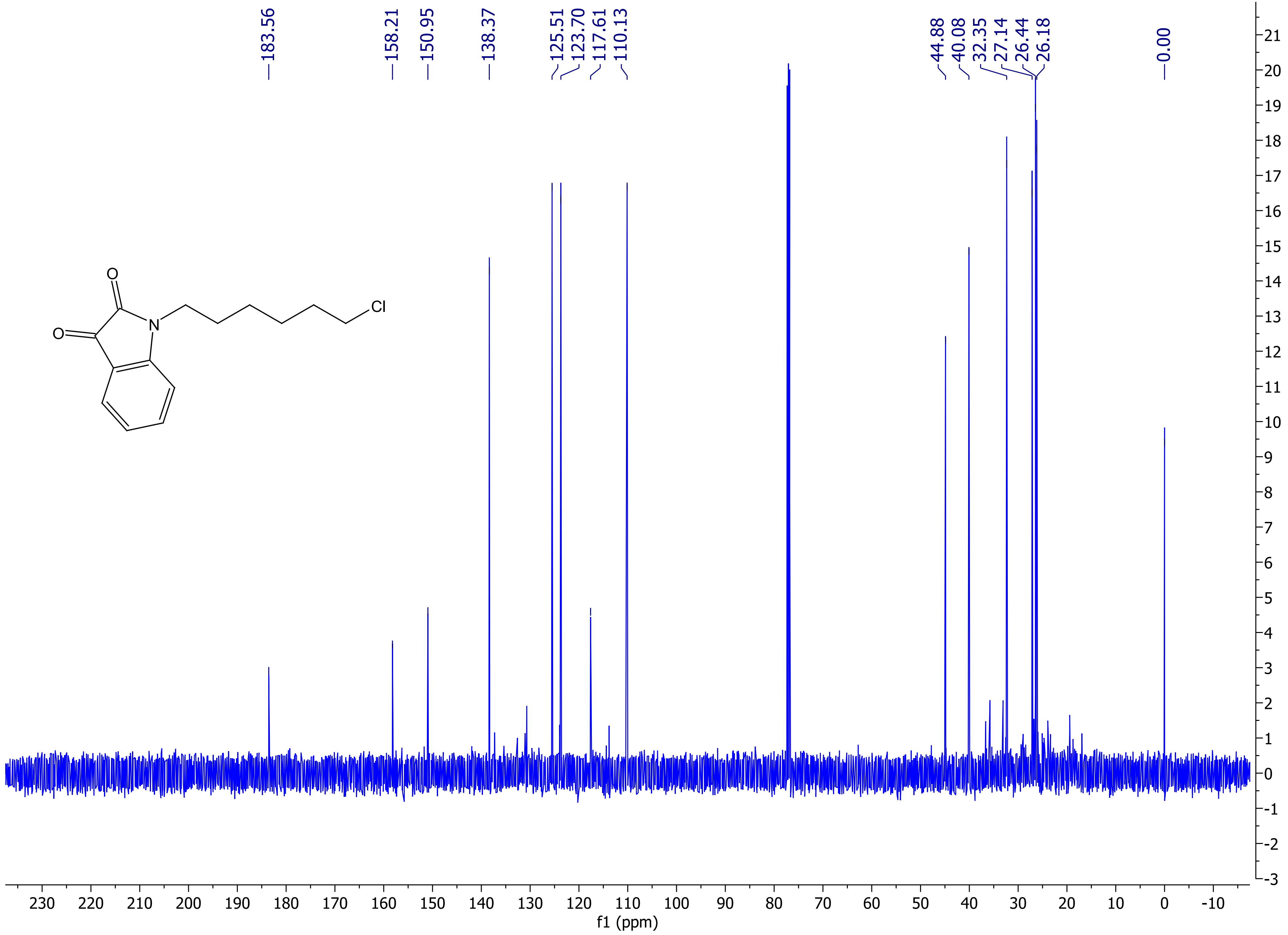
183.56
158.21
150.95
138.37
125.51
123.70
117.61
110.13
44.88
40.08
32.35
27.14
26.44
26.18
0.00
O
O
N
Cl
f1 (ppm)

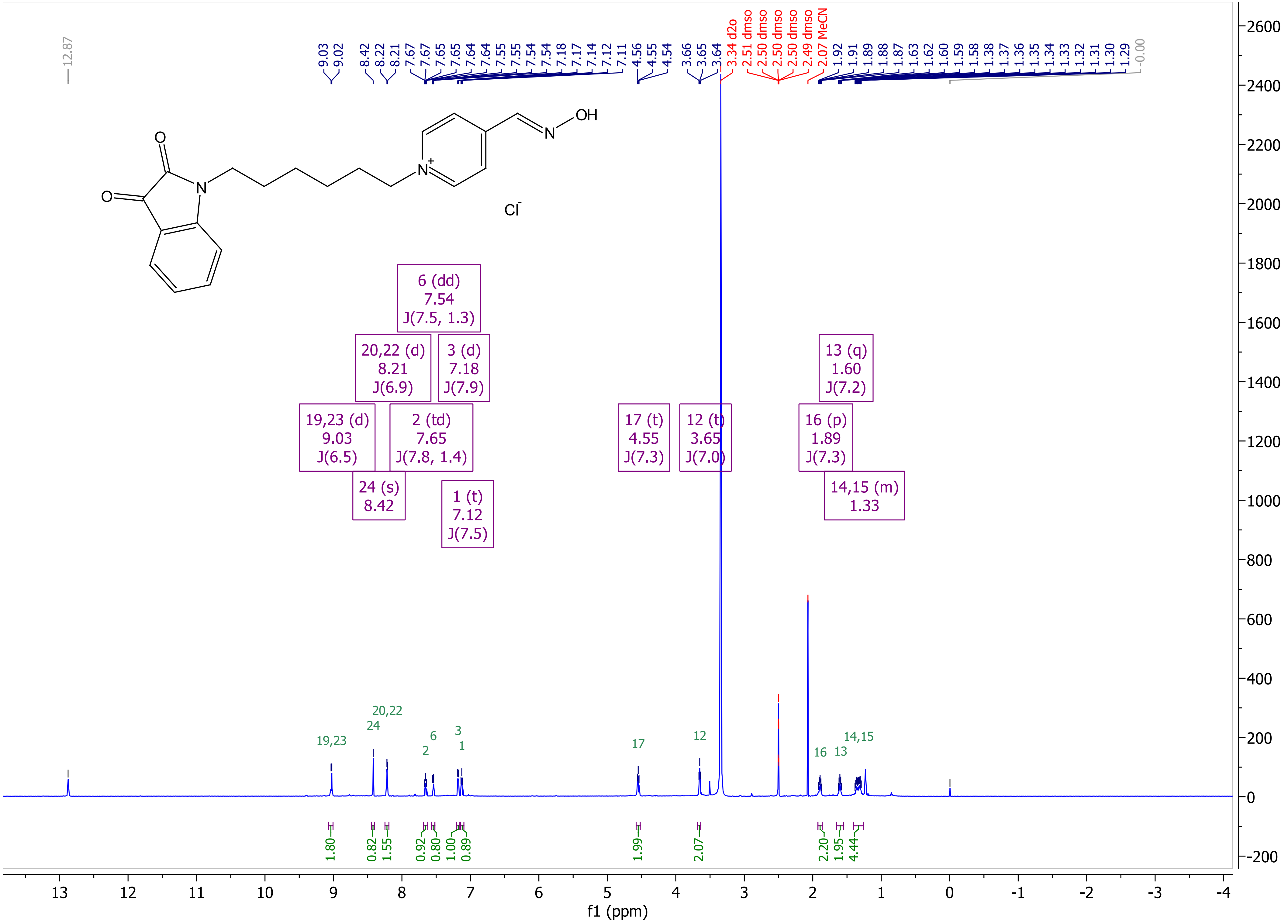

12.87
9.03
9.02
8.42
8.22
8.21
7.67
7.67
7.65
7.65
7.64
7.64
7.55
7.55
7.54
7.54
7.18
7.17
7.14
7.12
7.11
4.56
4.55
4.54
3.66
3.65
3.64
3.34 d2o
2.51 dmso
2.50 dmso
2.50 dmso
2.50 dmso
2.49 dmso
2.07 MeCN
1.92
1.91
1.89
1.88
1.87
1.63
1.62
1.60
1.59
1.58
1.38
1.37
1.36
1.35
1.34
1.33
1.32
1.31
1.30
1.29
-0.00
O
O
N
N+
OH
N
Cl-
6 (dd) 7.54 J(7.5, 1.3)
20,22 (d) 8.21 J(6.9)
3 (d) 7.18 J(7.9)
13 (q) 1.60 J(7.2)
19,23 (d) 9.03 J(6.5)
2 (td) 7.65 J(7.8, 1.4)
17 (t) 4.55 J(7.3)
12 (t) 3.65 J(7.0)
16 (p) 1.89 J(7.3)
24 (s) 8.42
1 (t) 7.12 J(7.5)
14,15 (m) 1.33
19,23
20,22
24
6
2
3
1
17
12
16
13
14,15
1.80
0.82
1.55
0.92
0.80
1.00
0.89
1.99
2.07
2.20
1.95
4.44
f1 (ppm)

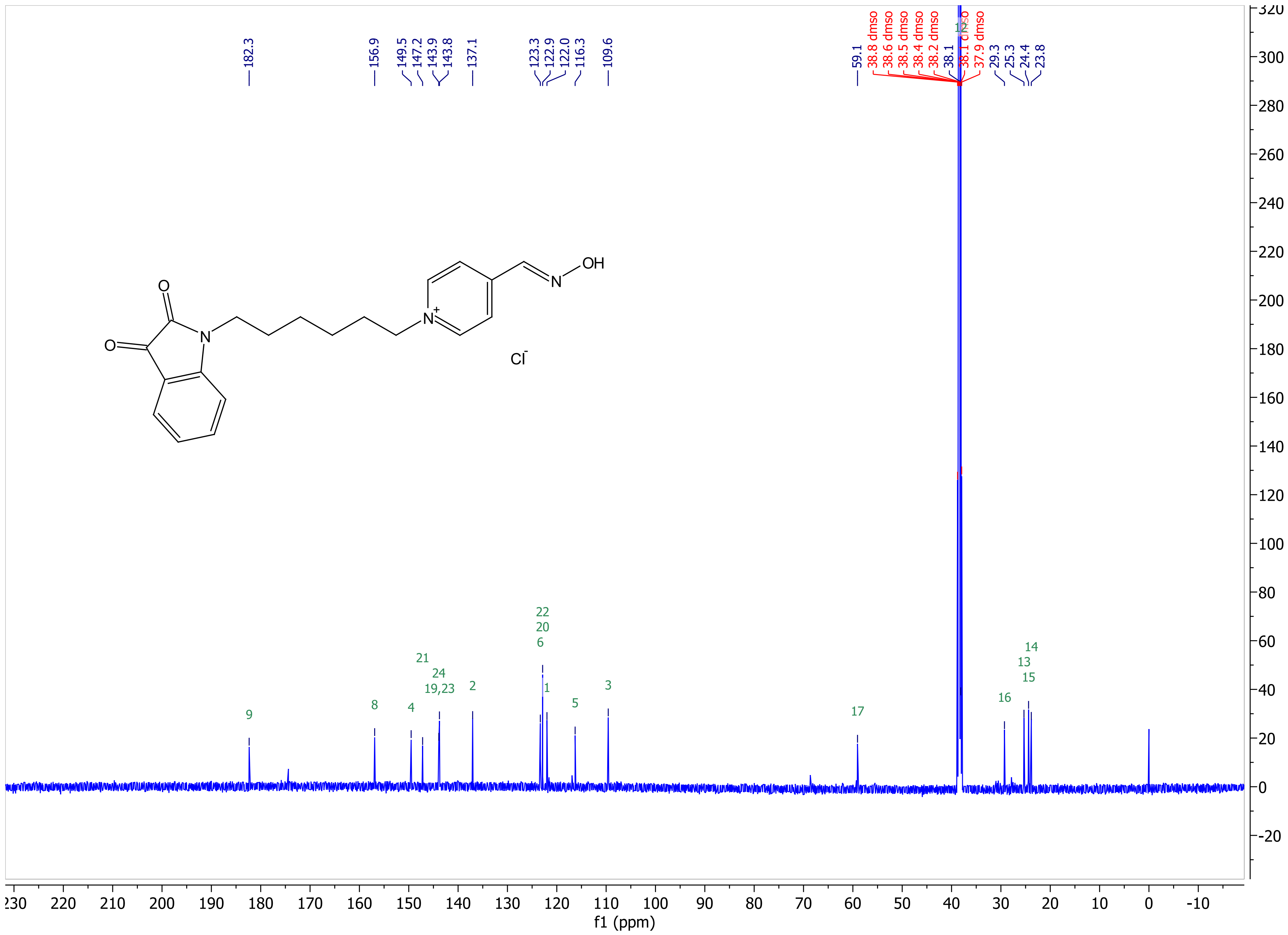
182.3
156.9
149.5
147.2
143.9
143.8
137.1
123.3
122.9
122.0
116.3
109.6
59.1
38.8 dmso
38.6 dmso
38.5 dmso
38.4 dmso
38.2 dmso
38.1
38.1 dmso
37.9 dmso
29.3
25.3
24.4
23.8
O
O
N
N+
OH
N
Cl-
9
8
4
21
24
19,23
2
22
20
6
1
5
3
17
16
14
13
15
f1 (ppm)

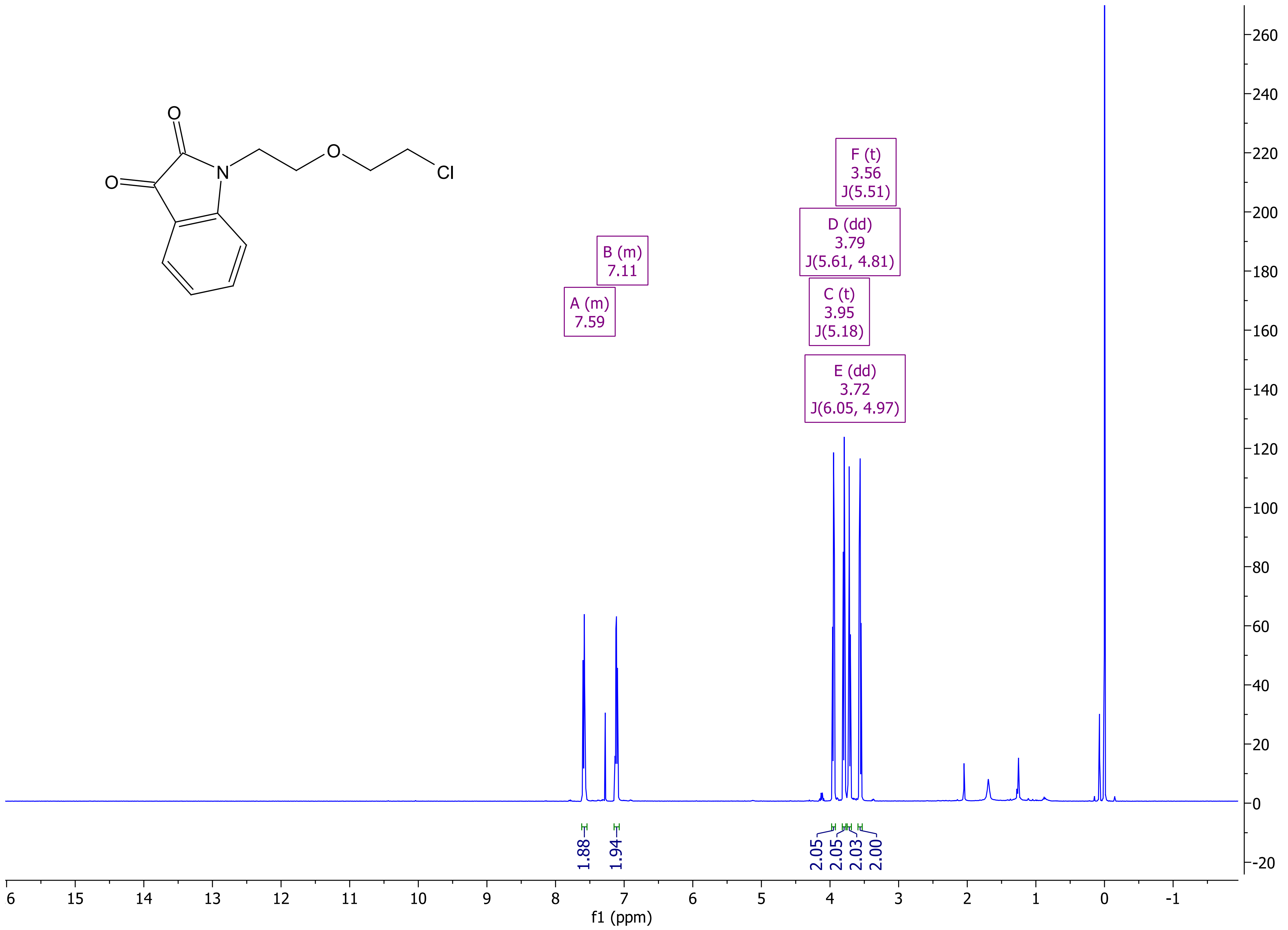

O
O
N
O
Cl
A (m)
7.59
B (m)
7.11
C (t)
3.95
J(5.18)
D (dd)
3.79
J(5.61, 4.81)
E (dd)
3.72
J(6.05, 4.97)
F (t)
3.56
J(5.51)
1.88
1.94
2.05
2.05
2.03
2.00
f1 (ppm)

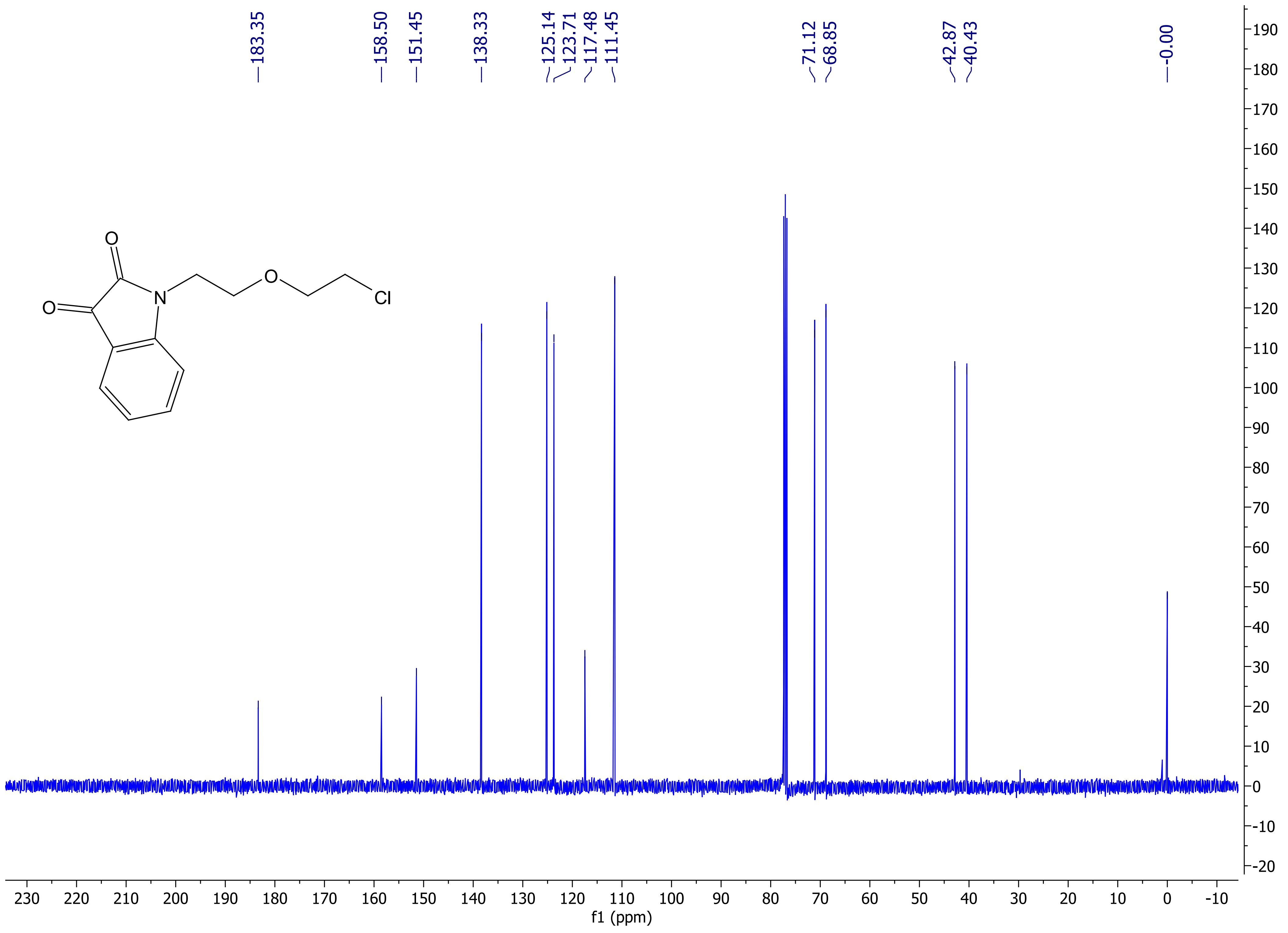

183.35
158.50
151.45
138.33
125.14
123.71
117.48
111.45
71.12
68.85
42.87
40.43
-0.00
O
O
N
O
Cl
f1 (ppm)

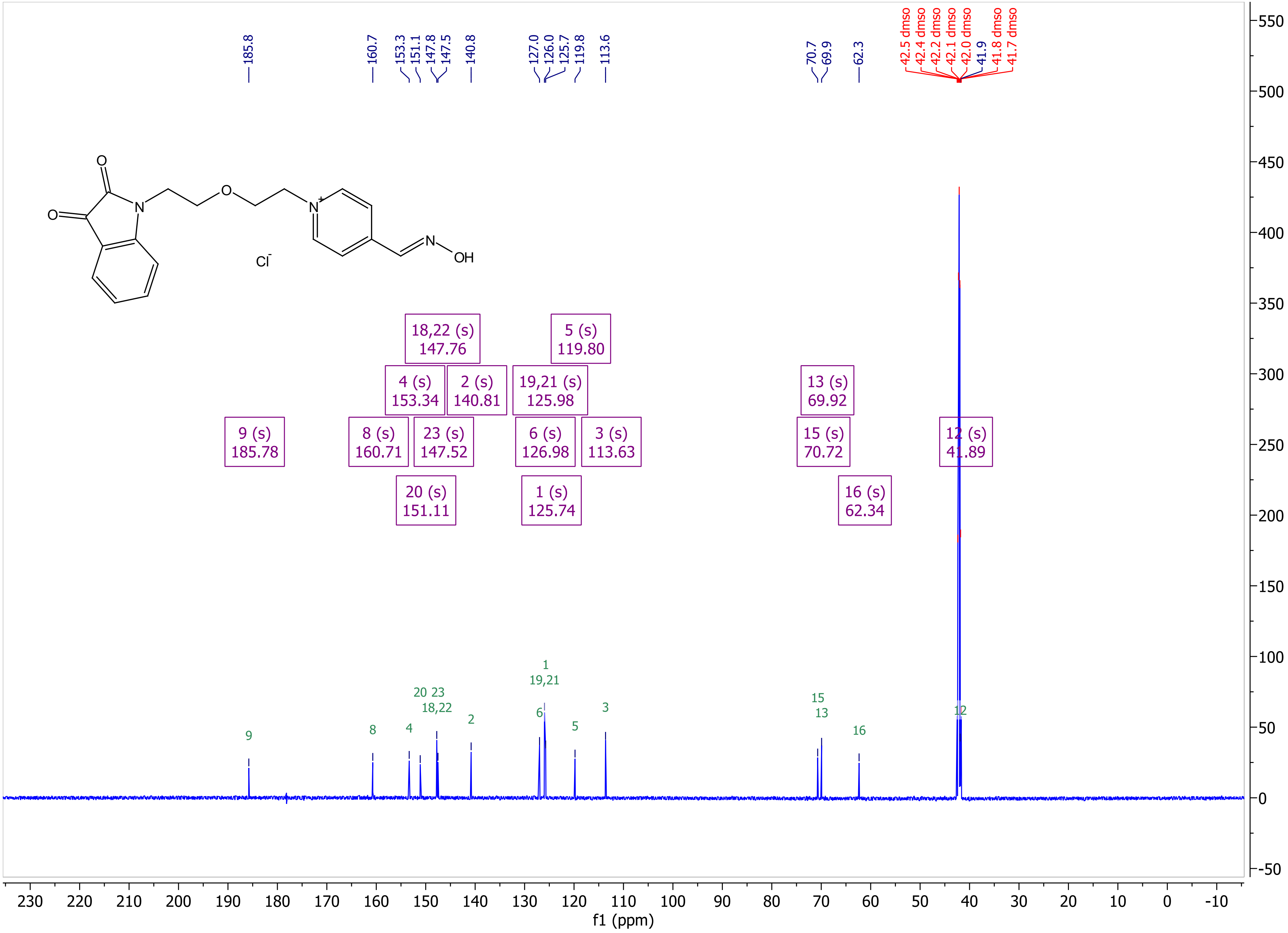
185.8
160.7
153.3
151.1
147.8
147.5
140.8
127.0
126.0
125.7
119.8
113.6
70.7
69.9
62.3
42.5 dmso
42.4 dmso
42.2 dmso
42.1 dmso
42.0 dmso
41.9
41.8 dmso
41.7 dmso
O
O
N
O
N+
N
OH
Cl-
18,22 (s) 147.76
5 (s) 119.80
4 (s) 153.34
2 (s) 140.81
19,21 (s) 125.98
13 (s) 69.92
9 (s) 185.78
8 (s) 160.71
23 (s) 147.52
6 (s) 126.98
3 (s) 113.63
15 (s) 70.72
12 (s) 41.89
20 (s) 151.11
1 (s) 125.74
16 (s) 62.34
9
8
4
20 23
18,22
2
1
19,21
6
5
3
15
13
16
12
f1 (ppm)

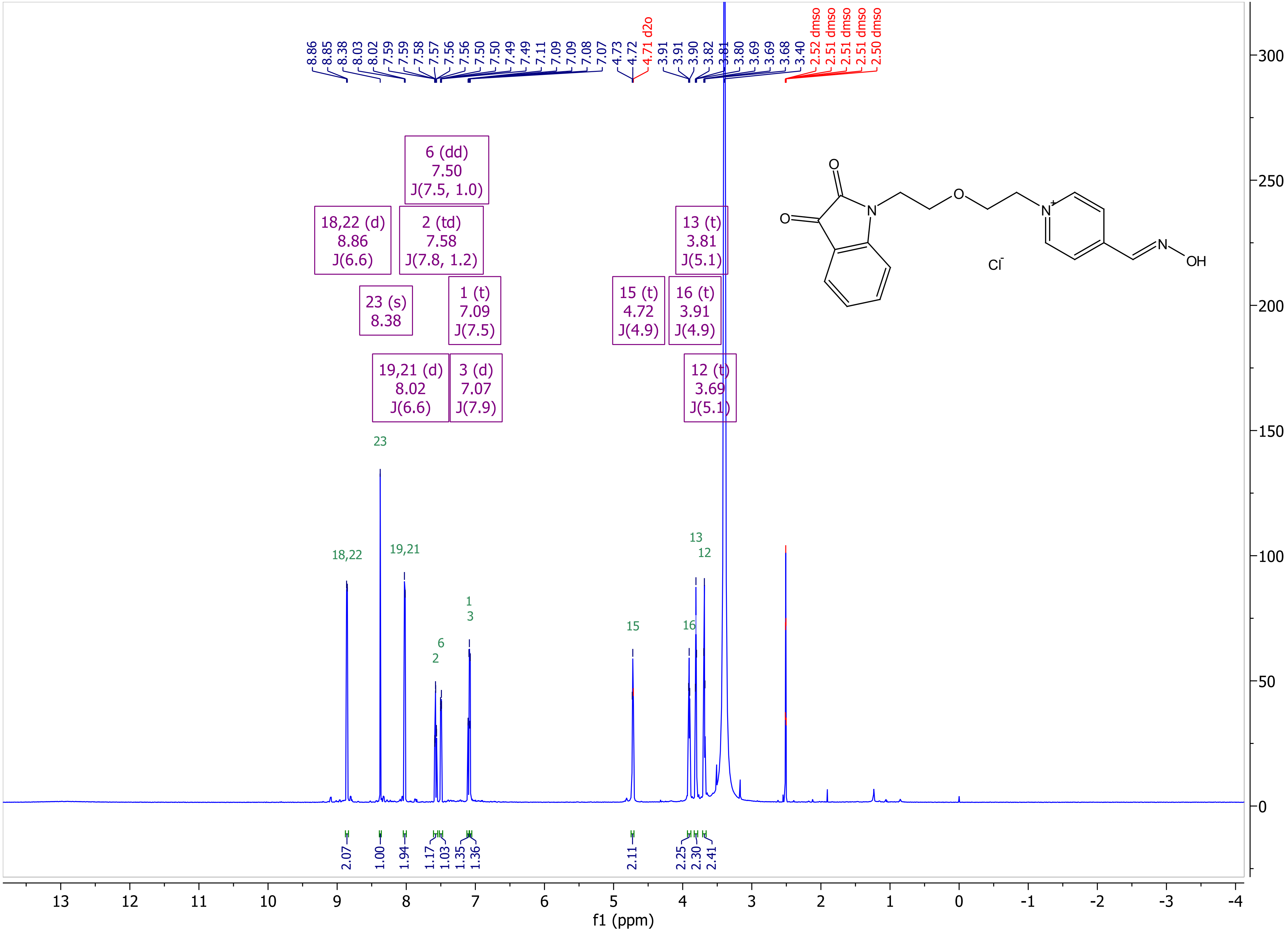

18,22 (d) 8.86 J(6.6)
23 (s) 8.38
19,21 (d) 8.02 J(6.6)
6 (dd) 7.50 J(7.5, 1.0)
2 (td) 7.58 J(7.8, 1.2)
1 (t) 7.09 J(7.5)
3 (d) 7.07 J(7.9)
15 (t) 4.72 J(4.9)
16 (t) 3.91 J(4.9)
13 (t) 3.81 J(5.1)
12 (t) 3.69 J(5.1)
4.71 d2o
2.52 dmso
2.51 dmso
2.51 dmso
2.51 dmso
2.50 dmso
Cl⁻
OH
f1 (ppm)

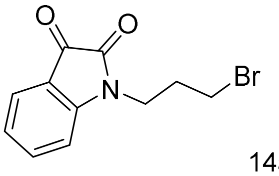

14a

1-(3-bromopropyl)indoline-2,3-dione

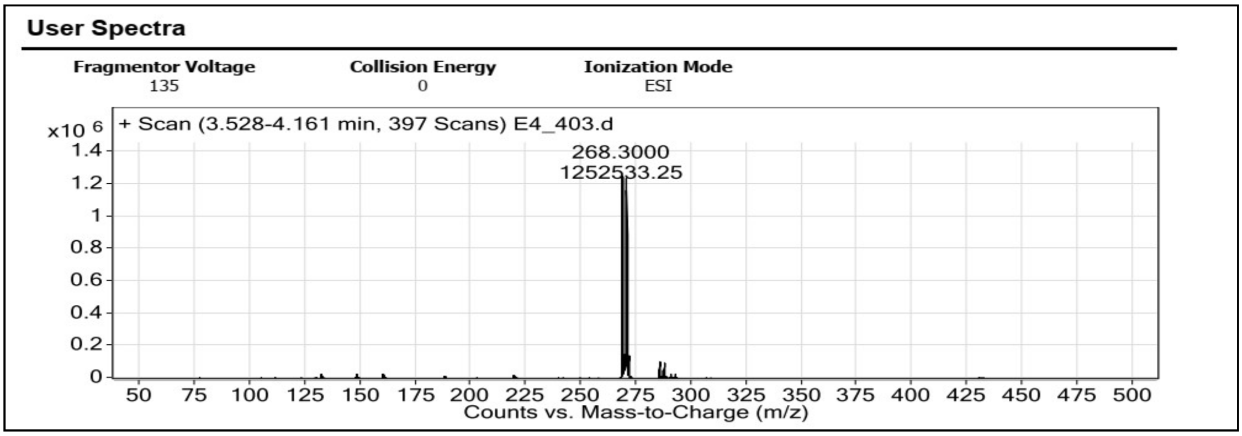

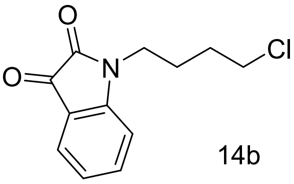

14b

1-(4-chlorobutyl)indoline-2,3-dione

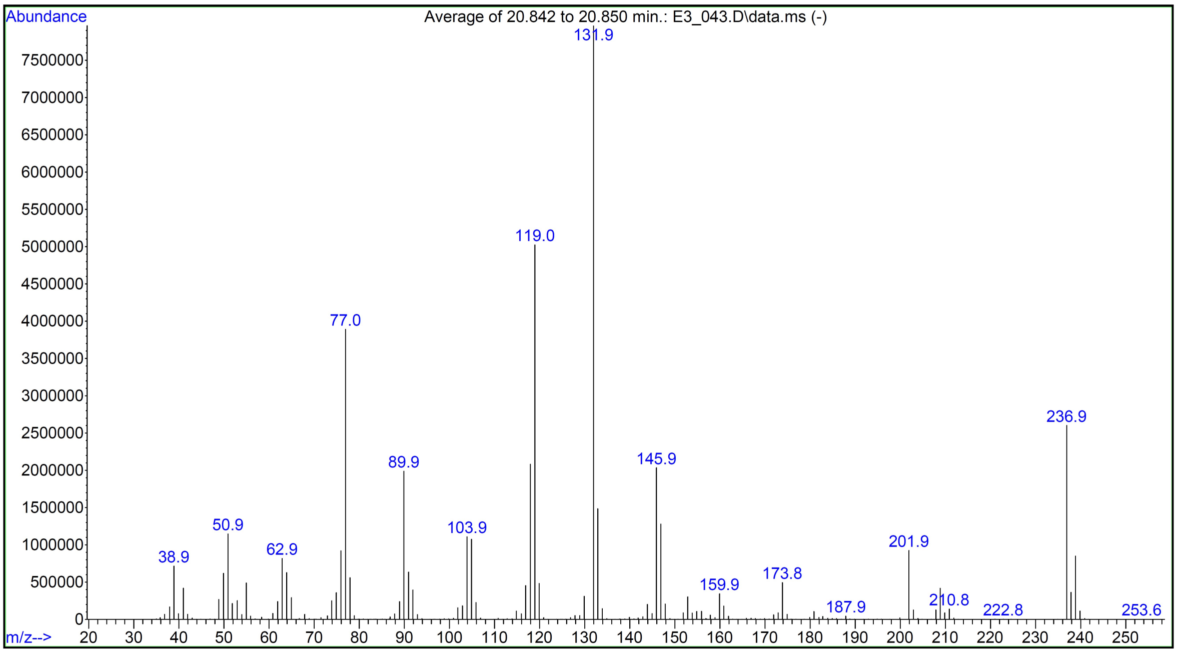

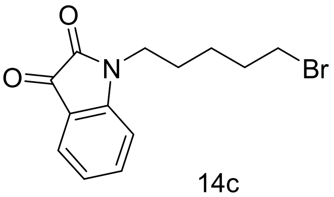

14c

1-(5-bromopentyl)indoline-2,3-dione

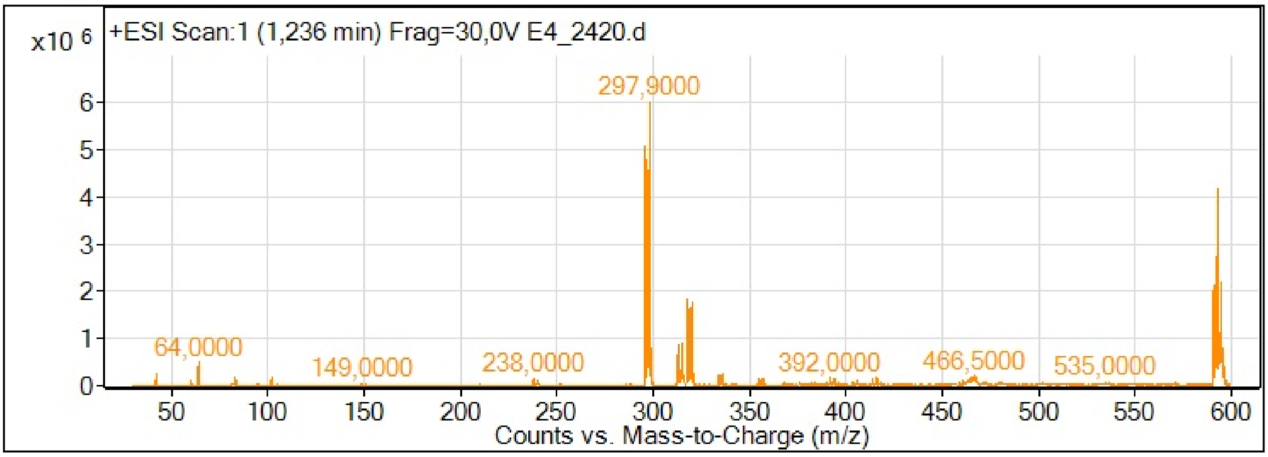

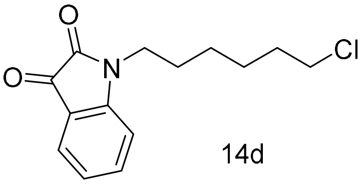

14d

1-(6-chlorohexyl)indoline-2,3-dione

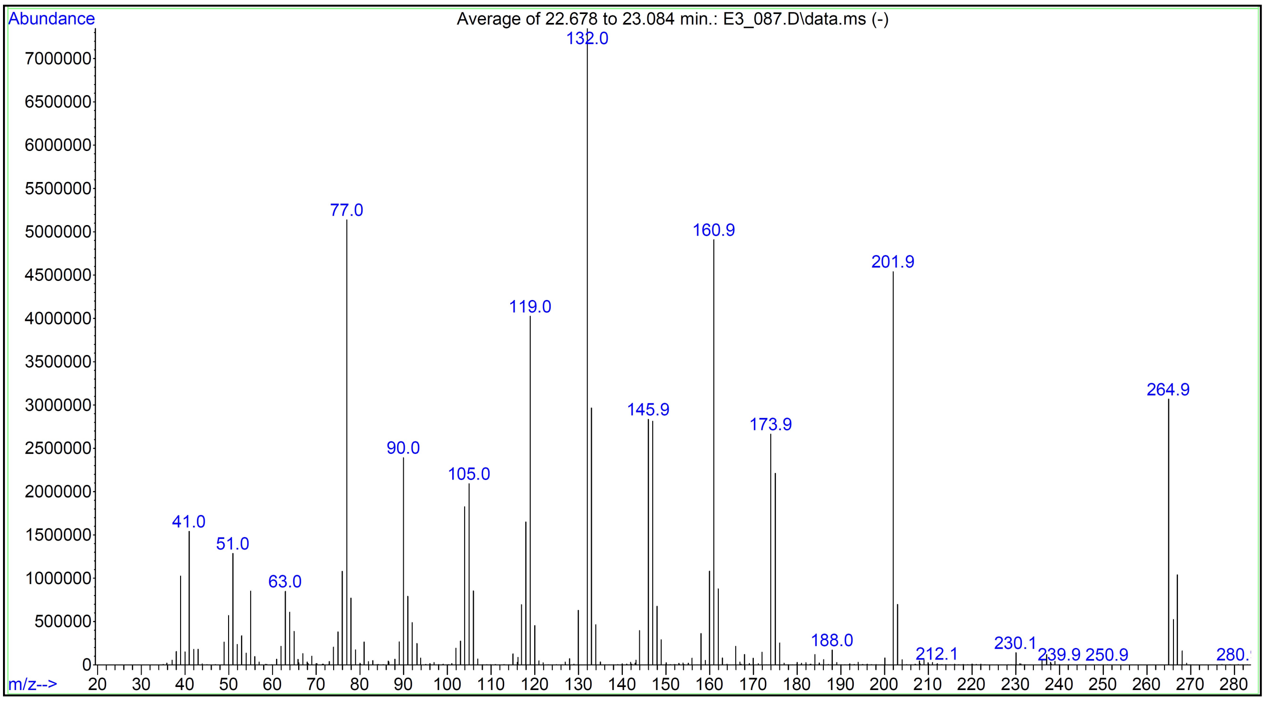

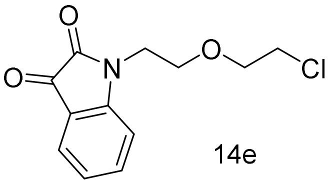

14e

1-(2-(2-chloroethoxy)ethyl)indoline-2,3-dione

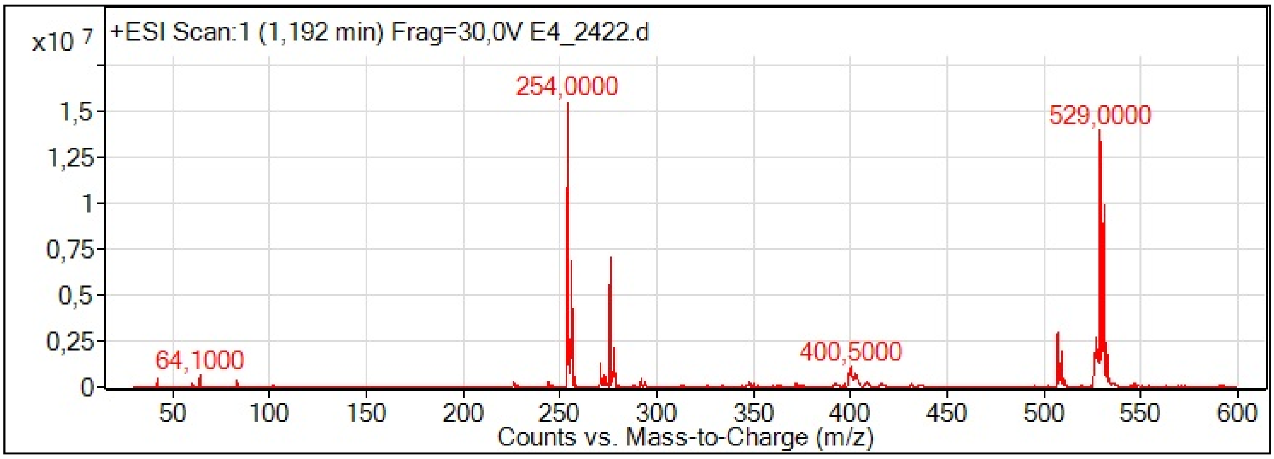

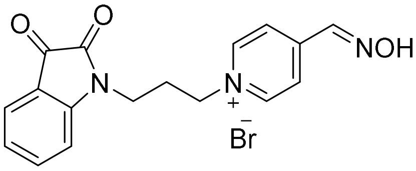

1-(3-(2,3-dioxoindolin-1-yl)propyl)-4-((hydroxyimino)methyl)pyridin-1-ium bromide (13a)

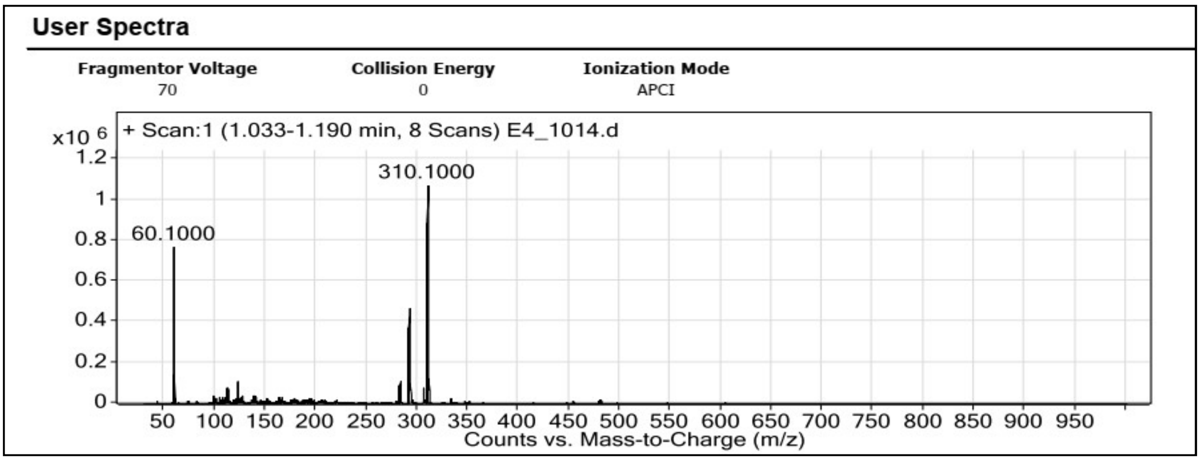

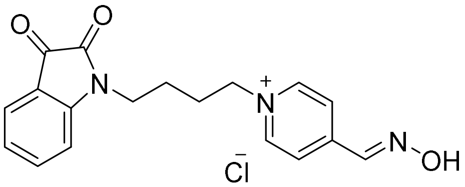

1-(4-(2,3-dioxoindolin-1-yl)butyl)-4-((hydroxyimino)methyl)pyridin-1-ium chloride (13b)

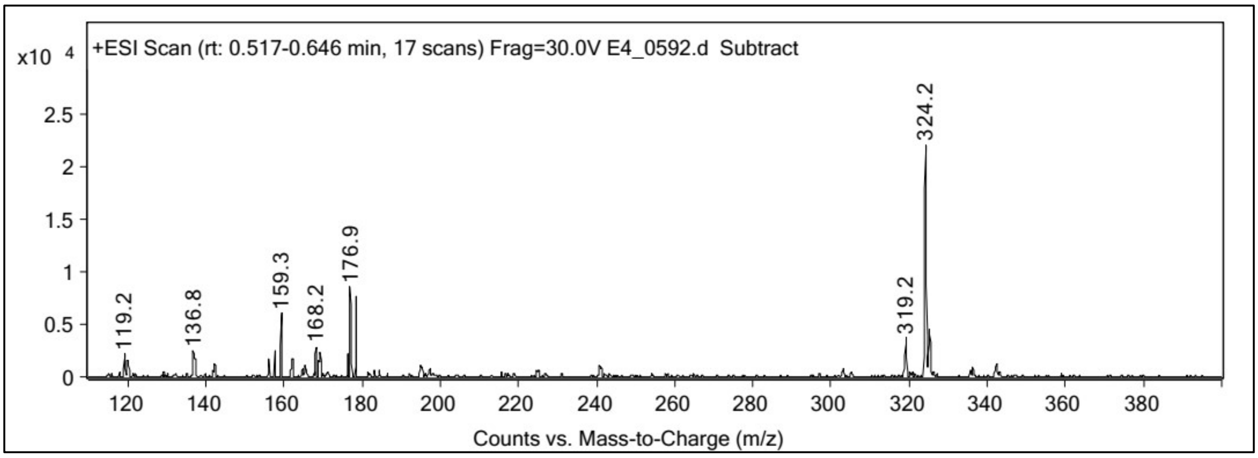

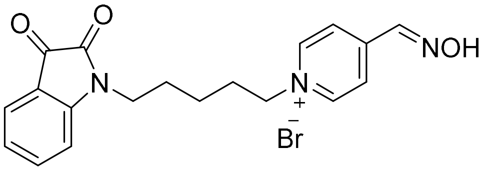

1-(5-(2,3-dioxoindolin-1-yl)pentyl)-4-((hydroxyimino)methyl)pyridin-1-ium bromide (13c)

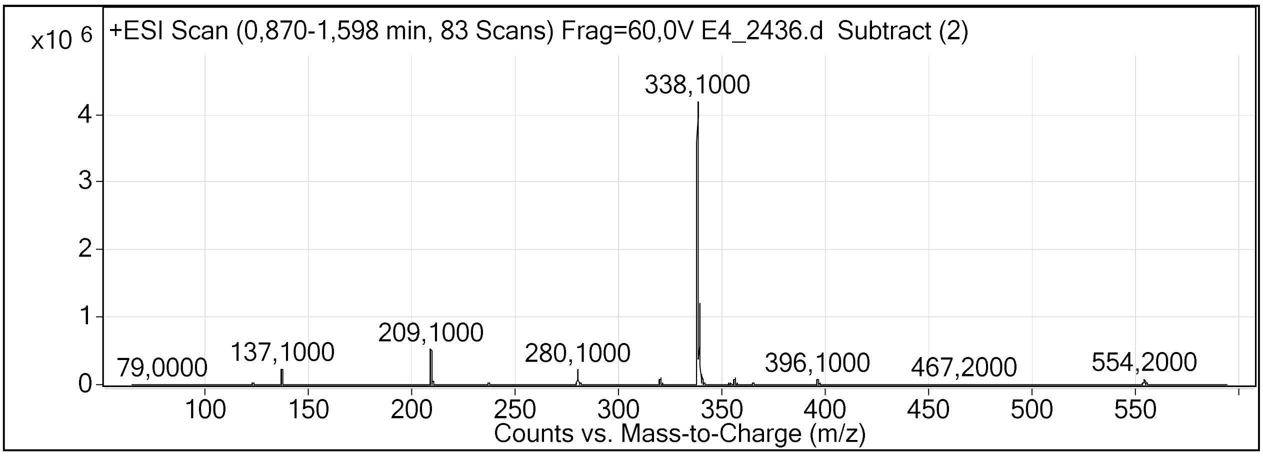

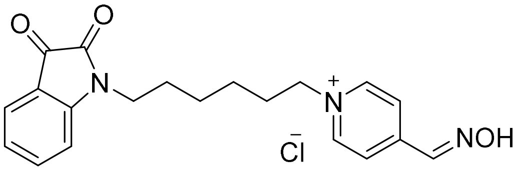

1-(6-(2,3-dioxoindolin-1-yl)hexyl)-4-((hydroxyimino)methyl)pyridin-1-ium chloride (13d)

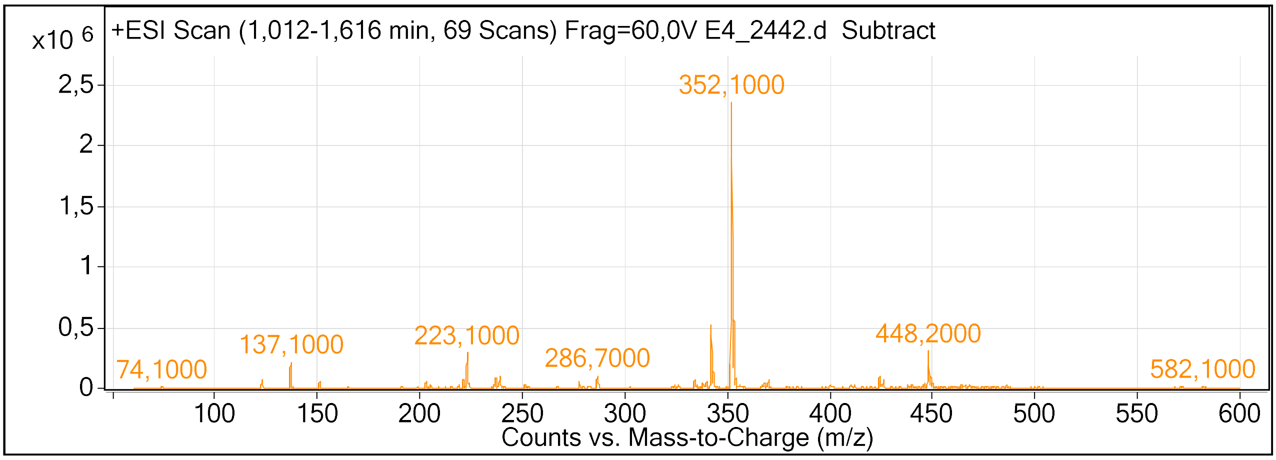

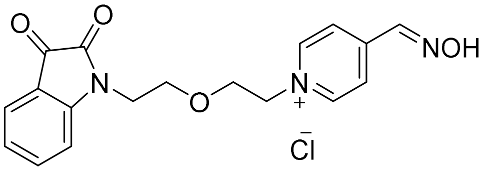

1-(2-(2-(2,3-dioxoindolin-1-yl)ethoxy)ethyl)-4-((hydroxyimino)methyl)pyridin-1-ium chloride (13e)

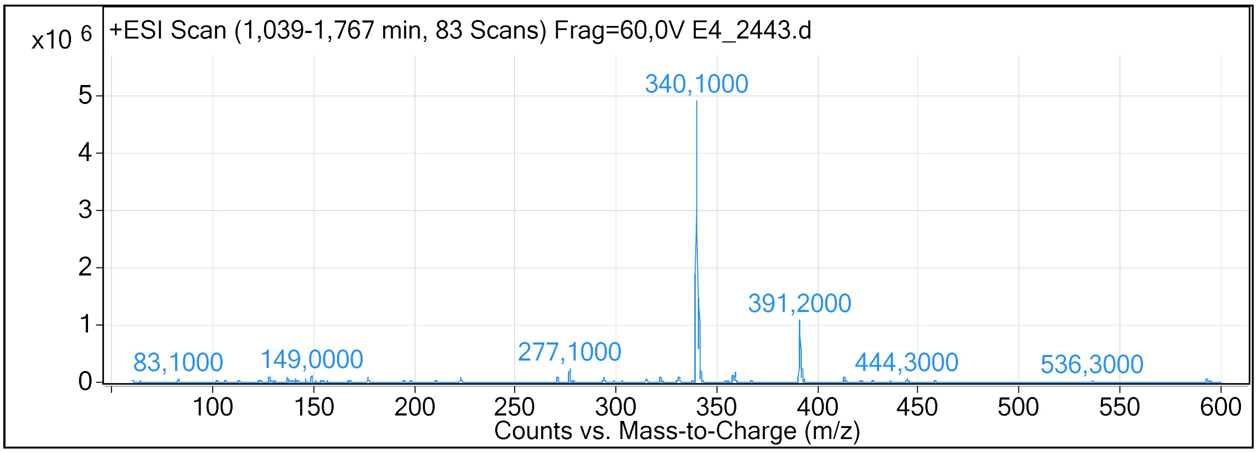

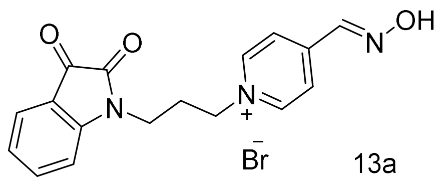
O
O
N
N
+
OH
N
Br

13a

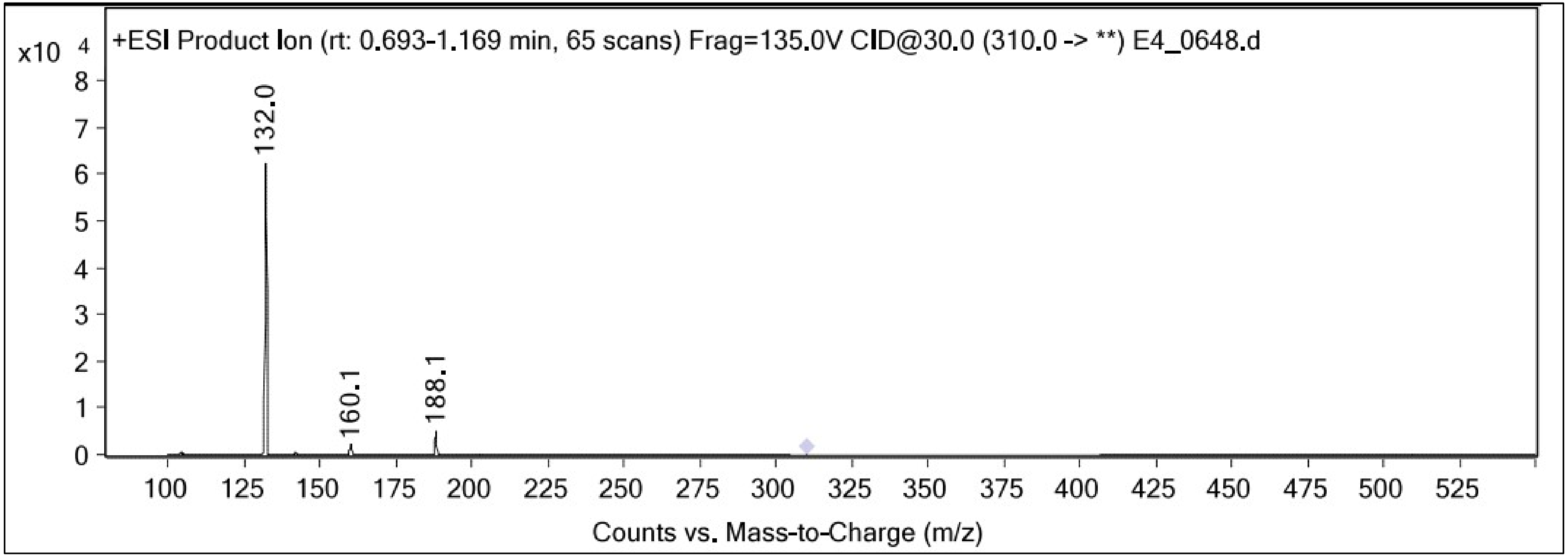
x10 4
+ESI Product Ion (rt: 0.693-1.169 min, 65 scans) Frag=135.0V CID@30.0 (310.0 -> **) E4_0648.d
132.0
160.1
188.1
Counts vs. Mass-to-Charge (m/z)

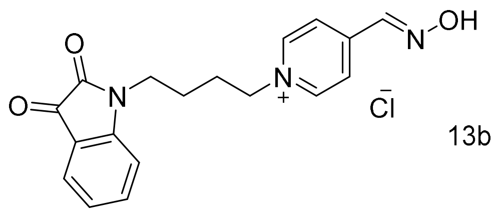

O
O
N
N
OH
Cl

13b

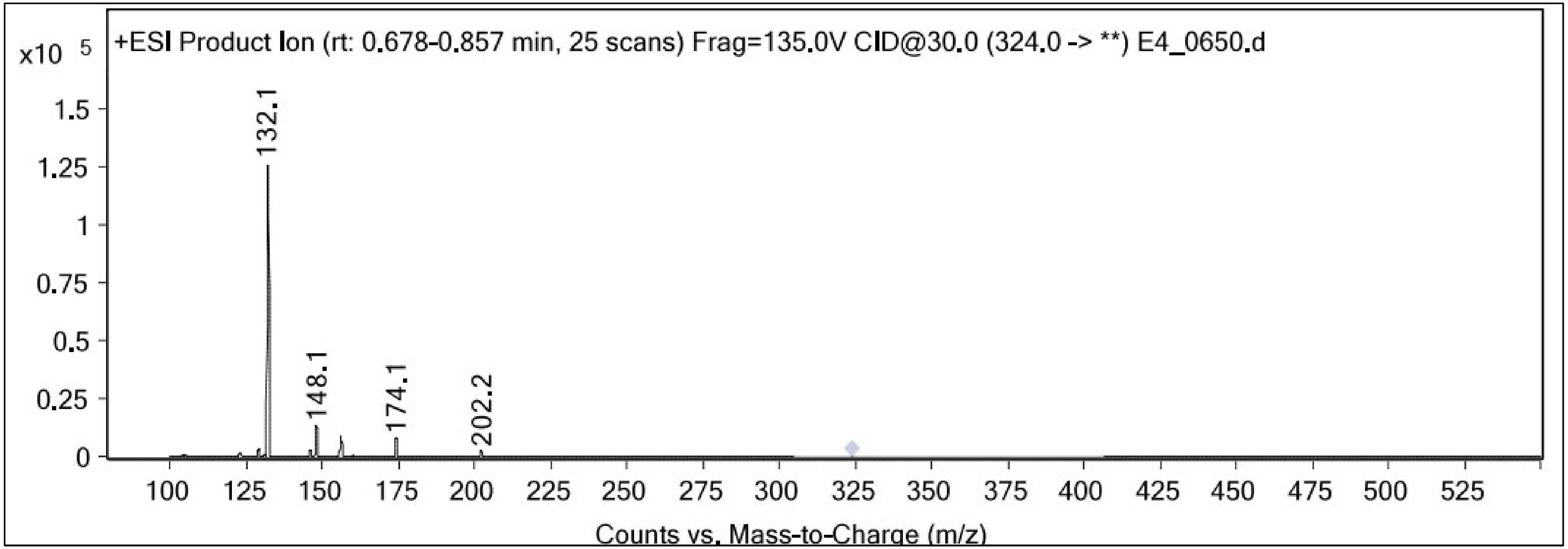

x10 5
+ESI Product Ion (rt: 0.678-0.857 min, 25 scans) Frag=135.0V CID@30.0 (324.0 -> **) E4_0650.d
132.1
148.1
174.1
202.2
1.5
1.25
1
0.75
0.5
0.25
0
100 125 150 175 200 225 250 275 300 325 350 375 400 425 450 475 500 525
Counts vs. Mass-to-Charge (m/z)

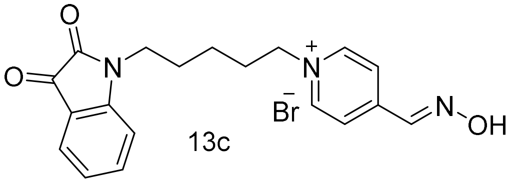
O
O
N
N
Br
N
OH

13c

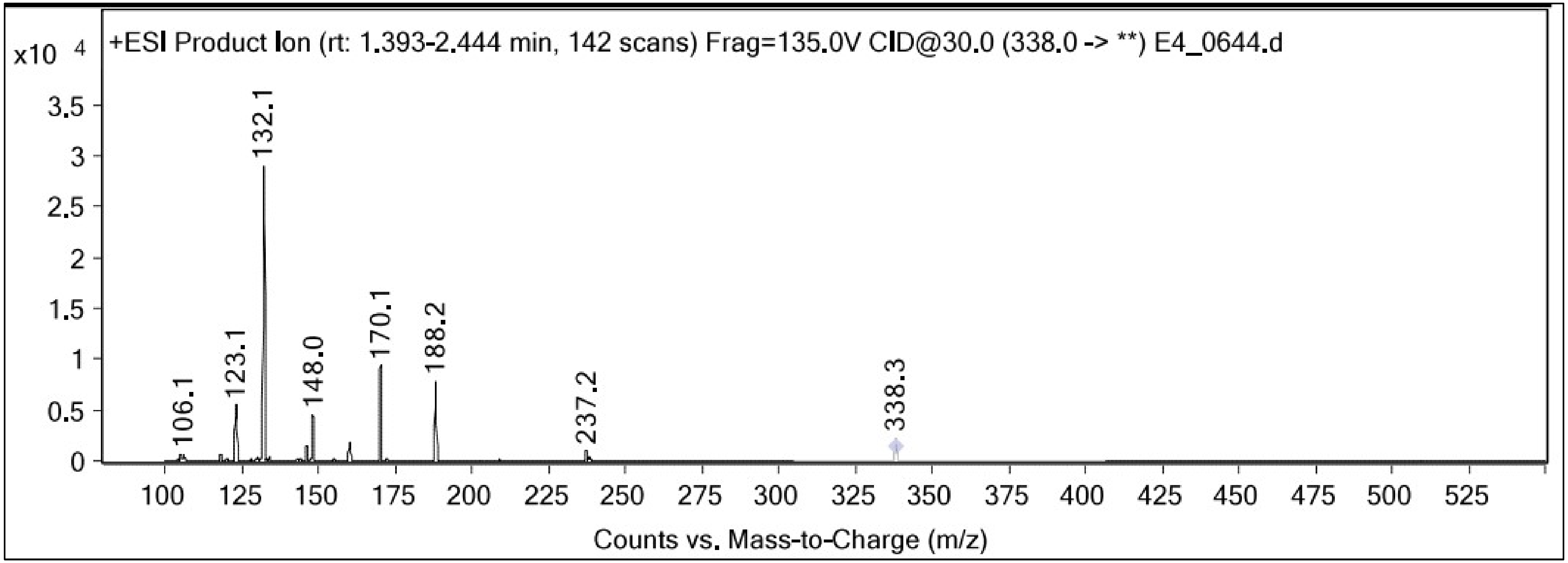
+ESI Product Ion (rt: 1.393-2.444 min, 142 scans) Frag=135.0V CID@30.0 (338.0 -> **) E4_0644.d
x10 4
3.5
3
2.5
2
1.5
1
0.5
0
132.1
123.1
106.1
148.0
170.1
188.2
237.2
338.3
100 125 150 175 200 225 250 275 300 325 350 375 400 425 450 475 500 525
Counts vs. Mass-to-Charge (m/z)

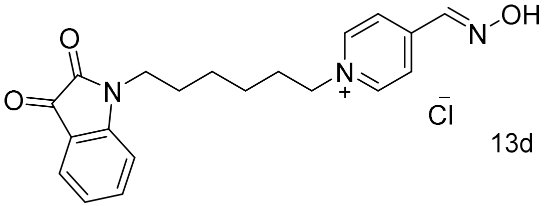

O
O
N
N
+
N
OH
Cl

13d

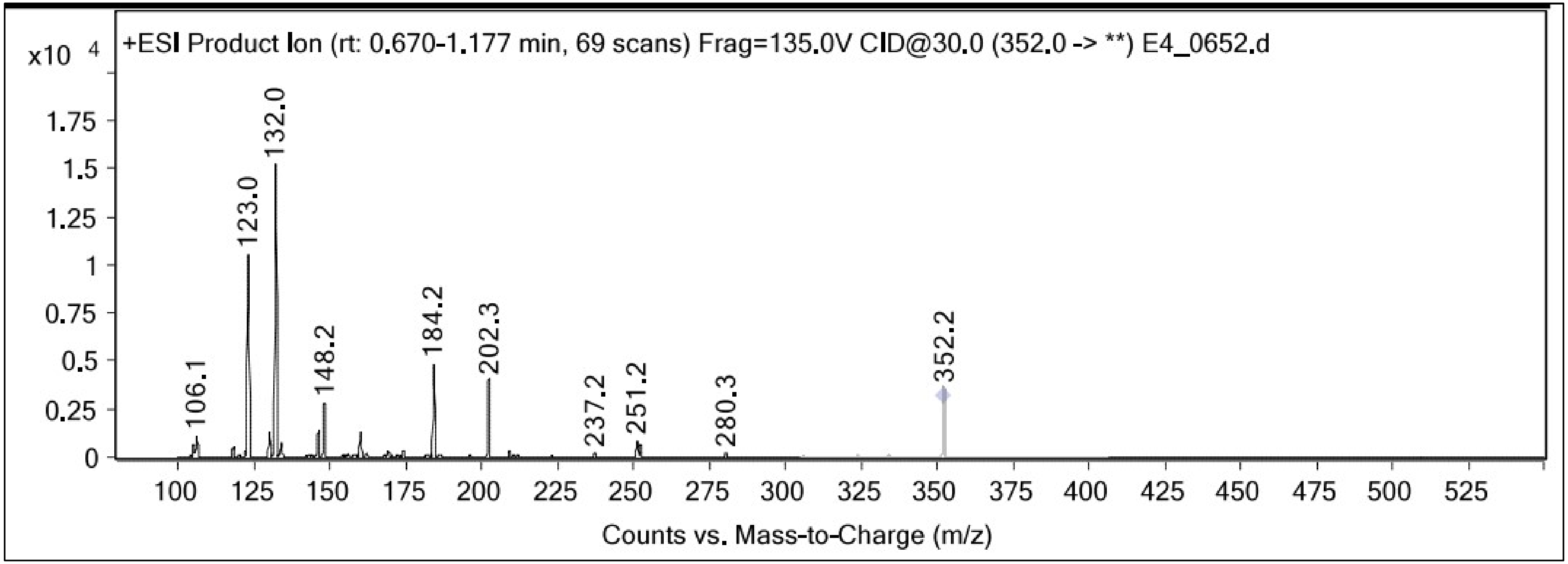

+ESI Product Ion (rt: 0.670-1.177 min, 69 scans) Frag=135.0V CID@30.0 (352.0 -> **) E4_0652.d
x10 4
1.75
1.5
1.25
1
0.75
0.5
0.25
0
106.1
123.0
132.0
148.2
184.2
202.3
237.2
251.2
280.3
352.2
100 125 150 175 200 225 250 275 300 325 350 375 400 425 450 475 500 525
Counts vs. Mass-to-Charge (m/z)

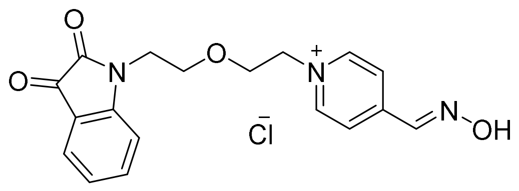
O
O
N
O
N
N
OH
Cl

13e

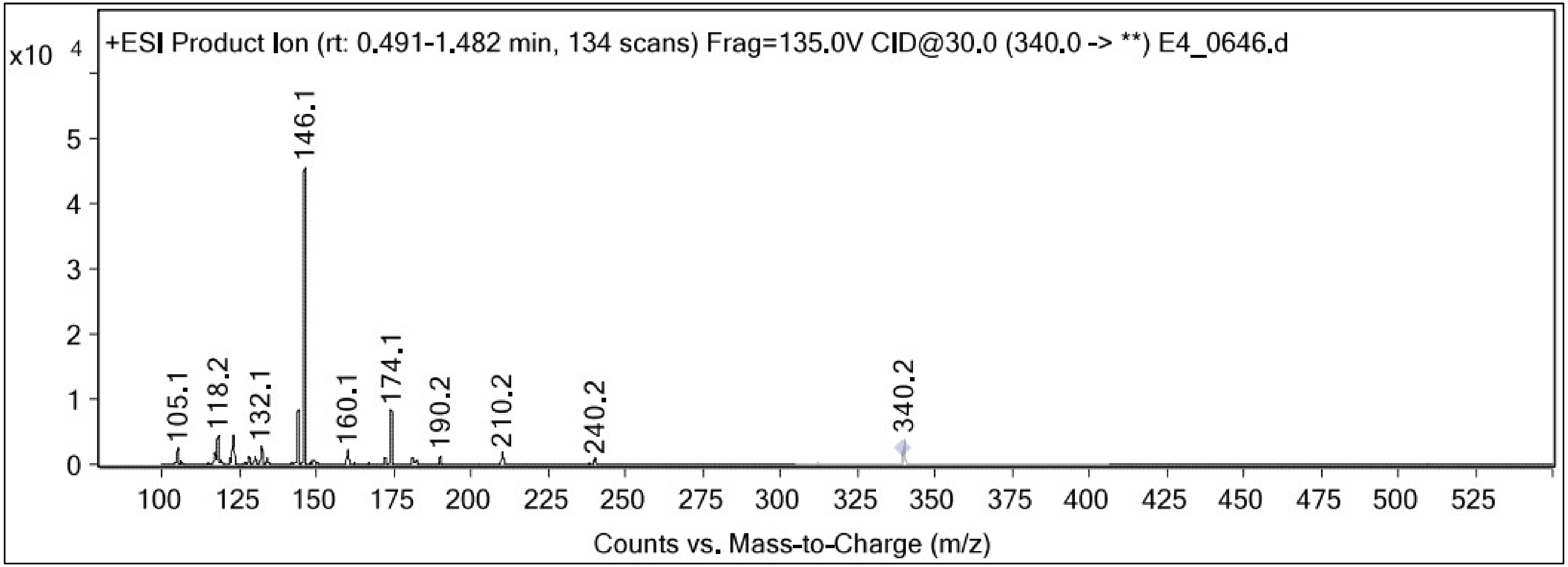
x10 4
+ESI Product Ion (rt: 0.491-1.482 min, 134 scans) Frag=135.0V CID@30.0 (340.0 -> **) E4_0646.d
146.1
105.1
118.2
132.1
160.1
174.1
190.2
210.2
240.2
340.2
Counts vs. Mass-to-Charge (m/z)
